# Combining Genome-Wide Gene Expression Analysis (RNA-seq) and a Gene Editing Platform (CRISPR-Cas9) to Uncover the Selectively Pro-oxidant Activity of Aurone Compounds Against *Candida albicans*

**DOI:** 10.3389/fmicb.2021.708267

**Published:** 2021-07-15

**Authors:** Fatmah M. Alqahtani, Scott T. Handy, Caleb L. Sutton, Mary B. Farone

**Affiliations:** ^1^Department of Biology, Middle Tennessee State University, Murfreesboro, TN, United States; ^2^Department of Chemistry, Middle Tennessee State University, Murfreesboro, TN, United States

**Keywords:** *Candida albicans*, drug discovery, mode of action, pro-oxidant activity, trehalose protection, aurone antifungal, sulfur amino acid protection

## Abstract

*Candida albicans* is the major fungal cause of healthcare-associated bloodstream infections worldwide with a 40% mortality rate. The scarcity of antifungal treatments due to the eukaryotic origin of fungal cells has challenged the development of selectively antifungal drugs. In an attempt to identify novel antifungal agents, aurones SH1009 and SH9051, as synthetically bioactive compounds, have been recently documented as anti-*Candida* agents. Since the molecular mechanisms behind the inhibitory activities of these aurones in *C. albicans* are unclear, this study aimed to determine the comprehensive cellular processes affected by these aurones and their molecular targets. Genome-wide transcriptional analysis of SH1009- and SH9051-treated *C. albicans* revealed uniquely repressed expression in different metabolic pathways, particularly trehalose and sulfur amino acid metabolic processes for SH1009 and SH9051, respectively. In contrast, the most commonly enriched process for both aurones was the up-regulation of RNA processing and ribosomal cleavages as an indicator of high oxidative stress, suggesting that a common aspect in the chemical structure of both aurones led to pro-oxidative properties. Additionally, uniquely induced responses (iron ion homeostasis for SH1009 and arginine biosynthesis for SH9051) garnered attention on key roles for the aurone functional groups. Deletion of the transcription factor for the trehalose biosynthesis pathway, Tye7p, resulted in an SH1009-resistant mutant, which also exhibited low trehalose content, validating the primary molecular target of SH1009. Aurone SH9051 uniquely simulated an exogenous supply of methionine or cysteine, leading to sulfur amino acid catabolism as evidenced by quantifying an overproduction of sulfite. Phenyl aurone, the common structure of aurones, contributed proportionally in the pro-oxidative activity through ferric ion reduction effects leading to high ROS levels. Our results determined selective and novel molecular mechanisms for aurone SH1009 and also elucidated the diverse cellular effects of different aurones based on functional groups.

## Introduction

*Candida albicans* is a fungal-commensal microorganism that normally grows as a member of the skin and mucosal microbiota of healthy individuals. However, it can evolve in association with human hosts as an opportunistic pathogen. The resulting candidiasis can range from superficial (thrush) to life-threatening bloodstream infections. Systemic infections principally develop in immune-compromised patients with mortality rates of near 40% (Brown et al., 2012; Kullberg and Arendrup, 2015). The striking ability of *C. albicans* to cause persistent infections is attributed to the reversible transition from the planktonic-yeast form to a hyphal form in order to build multicellular structures known as biofilms on abiotic or biotic surfaces which enable this pathogenic fungus to resist antifungals and overcome host defenses (Prasad, 2017).

The eukaryotic biological similarities between fungal and human cells obstruct the design and the development of antifungal drugs and limits their number to only five different chemical classes, including polyenes, echinocandins, azoles, pyrimidine analogs, and allylamines. The narrow range of targets for these available drugs and the resistances to them necessitate the need for new classes of antifungal agents (Prasad et al., 2019). Natural products that are derived from plants as secondary metabolites have been considered vital sources of potential antimicrobial compounds in the pharmaceutical industry in the last two decades. As plants evolved defenses against pathogenic fungi, the evolutionary pressure produced a high diversity of novel chemical skeletons by which to synthesize bioactive molecules with antifungal activity (Mishra and Tiwari, 2011). Aurones (2-benzylidene-1-benzofuran-3-one) are a subclass of flavonoids, widespread polyphenolic compounds in plants, with antifungal characteristic against *Candida* spp. (Sutton et al., 2017).

Our prior study showed that the bioactive aurone compound SH1009 exhibited selectively fungistatic-inhibitory activity against *Candida* spp. Additionally, mode-of-action screening using chemical genetic interaction in *Saccharomyces cerevisiae* indicated that SH1009 targets cell cycle-dependent organization of the actin cytoskeleton, providing informative clues about SH1009 antifungal activity, but this could not explain the selective toxicity of aurone SH1009 toward *Candida* spp. (Alqahtani et al., 2019). Despite the validation of chemical genetic interaction findings in the *C. albicans* SC5314 strain, the fundamental differences between *S. cerevisiae* and *C. albicans* physiology requires a target-identification approach in the major pathogenic yeast of our study *C. albicans*. These fundamental differences between *S. cerevisiae* and the pathogen include the ability of *C. albicans* to rapidly change its morphogenesis from yeast to true hyphae and differences in sensitivity and resistance to antifungal agents. Moreover, a comparison between *C. albicans* and *S. cerevisiae* genome sequences has shown that only about two-thirds of *Candida* genes have obvious orthologs in *Saccharomyces* genes (Prasad, 2017).

In this study, we further explore the antifungal activity of SH1009 in addition to another bioactive aurone compound, SH9051, which was previously documented for its inhibitory activity against *Candida* spp. and shares an identical core chemical structure with SH1009. However, instead of hydroxyl (-OH) and methoxy (-OCH_3_) functional groups, SH9051 contains a nitrogen (-N) atom in its B ring (Sutton et al., 2017). Also, a whole-genome transcriptional analysis post aurone SH1009 and SH9051 treatments of the *C. albicans* SC5314 strain was performed using RNA-seq technique. Accordingly, a reverse genetic approach was also applied to generate *C. albicans* deletion strains in order to validate the repressed target pathway of each aurone. Quantitative biochemical assays for specific cellular products were performed to precisely detect the cellular effects of the functional groups in the activity of each aurone. The results of this work improve the concept of structure-activity relationship for the aurones, providing a molecular and biochemical basis toward a comprehensive understanding of the mechanisms beyond the antifungal activity of aurones against *C. albicans*.

## Results

### Antifungal Assessments of Aurones SH1009 and SH9051

**FIGURE 1:**
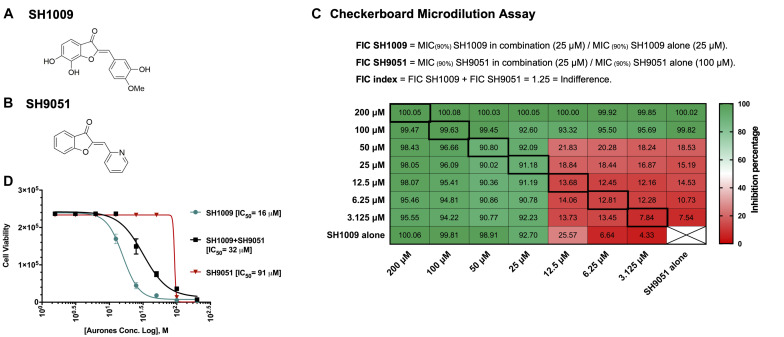
Antifungal assessments of aurones SH1009 and SH9051 against *C. albicans* SC5314 strain. **(A)** The chemical structures of aurones SH1009 and **(B)** SH9051. **(C)** The checkerboard microdilution assay in 96-well plate format. Each cell represents the percent inhibition of *C. albicans* (0.5–2.5 × 10^3^ CFU/mL) growth during aurone SH9051 and SH1009 treatments (3.125–200 μM) in RPMI-1640 medium for 24 h at 35^o^C either in combination or alone. **(D)** Graphing the non-linear regression of the cell viability readings based on the fluorescence intensity of PrestoBlue reagent using GraphPad Software to calculate the IC_50_ (concentration causing 50% growth inhibition) values of SH1009, SH9051, and SH1009 + SH9051 combination after transforming the molar concentrations of the aurones into logarithmic form. The cell viability readings of SH1009 + SH9051 combination were obtained from the bolded squares on the checkerboard plate.

Aurones SH1009 and SH9051 possess an identical core of their chemical structure with different functional groups, but they exhibited different half maximal inhibitory concentration (IC_50_) values against *C. albicans* (Figures 1A,B). Therefore, the potential of any synergistic interaction of their combination was investigated using a classical checkerboard microdilution assay. The fractional inhibitory concentration index (FICI) value between SH1009 and SH9051 was observed to be an indifferent effect (FICI = 1.25) against *C. albicans* SC5314 (Figure 1C). The growth inhibition by both compounds in combination was more than 90% at the concentrations of 25 μM for SH009 and 100 μM for SH9051, which is in the zone of indifference, suggesting different modes of toxicity (Figure 1D).

Cytotoxic effects of aurone SH1009 in mammalian cells were reported previously, indicating a selective antifungal toxicity toward the pathogenic *C. albicans* SC5314 strain (Alqahtani et al., 2019). The cytotoxic effects of aurone SH9051 on the same human cell lines (THP-1, HepG2, and A549) was carried out to examine the selectivity toward fungal cells. Supplementary Table 1 shows the cytotoxic concentrations of SH9051 that caused 50% loss of cell viability for each cell line, with SH9051 showing high CC_50_ values for each cell line. Since the IC_50_ of aurone SH9051 against *C. albicans* was 91.05 μM, low selective index values were obtained for THP-1 and HepG2 cells, but the difference between the CC_50_ of A549 cells and IC_50_ of fungal cells was two-fold. Conversely, aurone SH1009 demonstrated highly selective toxicity toward *C. albicans* cells. The concentration of 25 μM of SH1009 caused significant inhibition of *C. albicans* growth (>90% inhibition) while the cell viabilities for the human cells remained high at this concentration (Alqahtani et al., 2019). Here, a concentration of 100 μM of SH9051 showed the same inhibitory effects on *C. albicans* as 25 μM of SH1009, although this concentration of SH9051 decreased cell viabilities for the human cell lines (Supplementary Figure 1). These disparities in inhibitory concentrations and the indifference in the checkerboard results suggest that aurones SH1009 and SH9051 could target different biological pathways. Therefore, genome-wide gene expression analyses of *C. albicans* cells in response to aurone treatments were performed.

### Transcriptional Analysis of SH1009- and SH9051-Treated *C. albicans* Cells

**FIGURE 2:**
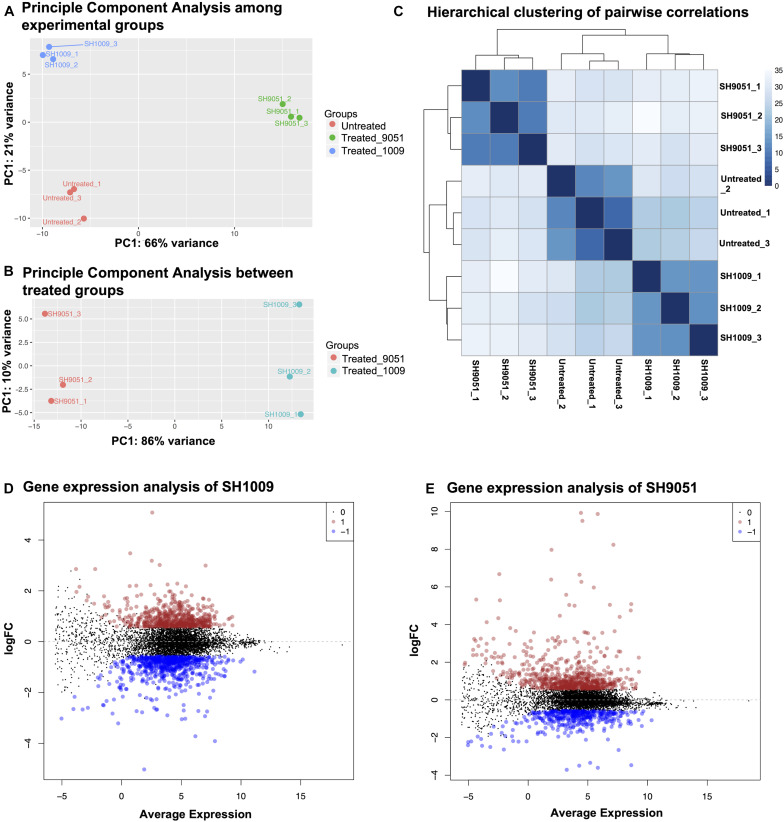
The reproducibility and differential expression analyses of RNA-seq data for SH1009-treated, SH9051-treated, and untreated *C. albicans* samples. **(A,B)** The principal component analysis plots of log-transformed counts where the *x*-axis represents the first dominant principal component (PC1) to measure the variance between experimental conditions and the *y*-axis represents the second dominant principal component (PC2) to measure the variance among the replicates within each sample. **(C)** Hierarchical clustering with heatmap to measure sample-to-sample distances where the depth of the blue color indicates the correlation values. **(D,E)** The mean dot (MD) plots of the log (fold change ≥ 1.5) for statistically significant genes (FDR ≤ 0.05) that are up-regulated (red dots) and down-regulated (blue dots) after 1 h exposure to aurones SH1009 and SH9051, respectively.

The effect of aurone treatments on *C. albicans* SC5314 transcriptional responses was investigated after 1 h incubation with concentrations that caused 30% inhibition. A high quality of total RNAs (≥1.5 μg and an A260/A280 ratio ≥ 2.0) with high RNA integrity number (RIN ≥ 9.7) (Supplementary File 1) was recovered for SH1009-treated, SH9051-treated and untreated cells. Genome-wide transcriptional analysis was performed using transcriptome sequencing (RNA-seq). The pre-analysis of RNA-seq computational analyses is a major step to detect any technical biases that may alter the biological differences. Therefore, diagnostic plots showed high-quality RNA-seq data regarding the alignment accuracy, sequencing depth, and filtered and normalized counts distribution (Supplementary Figure 2). The reproducibility among the conditional groups and within technical replicates demonstrated different gene expression profiles with no impact of batch effects. Notably, the two-dimensional plot of principal component analysis showed a distinct separation with greater variability between experimental groups by the first component, indicating that the treatment with aurones was the major source of differences (Figure 2A). In addition, the variability of gene expression profiles of SH1009-treated and SH9051-treated cells was increased to 86% variance when comparing these profiles to each other only, suggesting different modes of action for different aurones and supporting the checkerboard assay results (Figure 2B). The hierarchical clustering with pairwise correlations showed similar clustering that identified three groups of samples based on the treatment with dramatically different patterns of transcriptional profiles (Figure 2C). Treatment with SH1009 and SH9051 resulted in 1249 and 1085 differentially expressed genes between treated and untreated samples (*P* (FDR) ≤ 0.05 and fold change ≥ 1.5), respectively. A total of 585 genes were up-regulated, and 664 genes were down-regulated upon SH1009 treatment, whereas 689 genes were up-regulated, and 396 genes were down-regulated upon SH9051 treatment (Supplementary Tables 2, 3) compared to their regulation in *C. albicans* under untreated conditions (Figures 2D,E).

**FIGURE 3:**
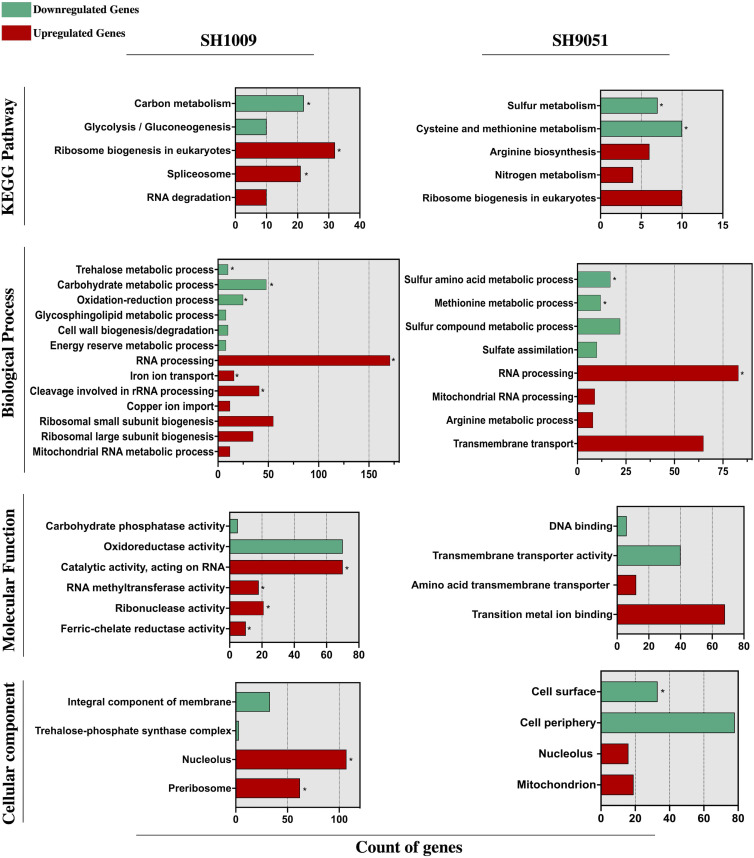
Functional enrichment analysis of KEGG pathway and gene ontology (biological process, molecular function, and cellular component) categories of significantly differential genes (FDR ≤ 0.05 and fold change ≥ 1.5) down- and up-regulated in response to aurones SH1009 and SH9051 treatment. TermFinder and FungiDB were used to find the significantly enriched KEGG/GO terms using GO categories based on a hypergeometric testing in the *C. albicans* SC5314 annotation with FRD ≤ 0.05 as a cutoff significant value. Green and red bars represent the number of respective down- and up-regulated genes that are clustered in each KEGG/GO term. GO terms within each group are listed according to increasing *P* values. Asterisks depict the highly significant KEGG/GO categories with *P* ≤ 0.0001.

To elucidate the transcriptional response of *C. albicans* SC5314 during aurone treatment, the induced and repressed genes were examined for clustered analysis of over-represented functionally annotated KEGG pathways and gene ontology (GO) terms in SH1009 and SH9051 transcriptomes. GO enrichment analyses showed that a large subset of down-regulated genes were significantly (*P* (FDR) values < 0.0001) enriched in response to cellular transporters and regulators associated with metabolic processes (carbon metabolism for SH1009 and sulfur metabolism for SH9051), suggesting that the exposure to chemically different structures of aurones robustly alters the nutrient availability in which the expression of genes that are involved in cellular metabolism and nutrient uptake are distinctively repressed. However, for up-regulated genes that were responsive to aurone treatment, GO enrichment analysis showed (*P* (FDR) < 0.0001) genes that are associated with RNA processing and ribosomal biogenesis in both aurone profiles in addition to uniquely induced responses for each aurone profile (iron ion homeostasis for SH1009 and arginine biosynthesis for SH9051), suggesting common aspects of detoxification mechanisms for *C. albicans* with specifically induced routes of adaptations in order to buffer the effects of particular nutrient deprivations (Figure 3).

### Different Metabolic Pathways Are Significantly Enriched by Growth With Different Aurones

**FIGURE 4:**
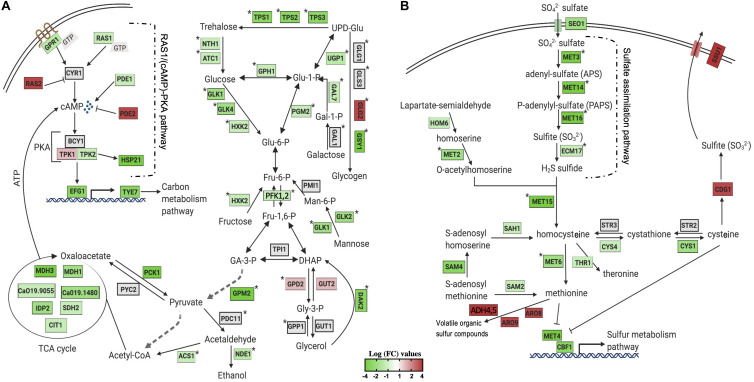
Visualization of the impact of aurone treatment on the central metabolism of significantly enriched KEGG pathways. The changes in gene expression in central carbon and sulfur metabolic pathways in response to SH1009 **(A)** and SH9051 **(B)** treatments. Detected genes are colored based on their values of log fold change, green and red colors indicate down- and up-regulated genes, respectively; a gray color indicates undetected genes. Pathway annotations and gene names are customized from different studies on *C. albicans* or based on their homology in *S. cerevisiae* and created with BioRender.com. Stars indicate genes that are directly activated by the major transcriptional activators Tye7p for carbon metabolism and Met4p for sulfur metabolism.

To obtain a clearer view of the sequential changes in gene expression patterns of *C. albicans* cells after SH1009 or SH9051 treatment, a gene regulatory network for the targeted pathway of each transcriptome profile is summarized in Figure 4A for SH1009 and Figure 4B for SH9051. As aforementioned, SH1009 caused a repression in the carbon metabolism pathway which is tightly regulated by the Ras1/cAMP/protein kinase A (PKA) pathway (Sabina and Brown, 2009). The glucose G-protein receptor gene *GPR1*, for which expression was down-regulated by −1.5-fold, controls the production of cAMP from ATP by activating the down-regulated GTPase gene *RAS1* (by −1.48-fold). In addition to Ras1, other key components of the Ras1/cAMP/PKA pathway were differentially expressed by the treatment. The up-regulated genes GTPase *RAS2* (by 2.2-fold) and phosphodiesterase *PDE2* (by 2.6-fold) primarily reduce the cellular level of cAMP, which is required for inactivation of the inhibitory subunit Bcy1 of PKA. The *PDE1* gene, which was down-regulated (by −1.49-fold), acts as the substrate for the PKA complex and provides negative feedback in carbohydrate signaling and regulation. When cAMP binds to the inhibitory subunit of the PKA complex, it releases its catalytic subunits, *TPK1*, which was up-regulated by 1.6-fold and is involved in stress response, and *TPK2*, which was down-regulated by −1.56-fold and involved in trehalose synthesis and iron uptake (Robertson et al., 2000; Lin and Chen, 2018). Activated PKA phosphorylates a series of transcription factors that control carbon metabolism, stress adaptation, cell cycle, and other functions, but the terminal point of the Ras1/cAMP-PKA pathway in *C. albicans* activation is the master transcription factor Efg1p. This transcription factor, for which expression was down-regulated by −2.4-fold, promotes yeast-to-hyphal transition through controlling carbon metabolism by directly regulating expression of the major glycolytic activator Tye7p that was down-regulated by −2.2-fold as well (Robertson et al., 2000).

The transcriptional factors in the down-regulated genes set for the SH1009-treated transcriptome were ranked based on the percentage of transcriptome regulation using the PathoYeastract database (Monteiro et al., 2017). The transcriptional regulator Tye7p was the top transcriptional factor that plays a key role in cellular response to SH1009 by regulating 44% of down-regulated genes. Tye7p regulates the down-regulated genes *TPS1* (by −2.3-fold), *TPS2* (by −2.5-fold), *TSP3* (by −2.4-fold), *ATC1* (by −1.5-fold), and *NTH1* (by −1.5-fold), all of which are involved in the most significantly enriched biological process in the SH1009 repressed transcriptome, trehalose metabolism (Askew et al., 2009). Moreover, genes that are associated with glucose, glycogen, and galactose biosynthesis were found dysregulated by SH1009 treatment, consequently resulting in a poor supply of carbohydrate, which is the preferred source of energy in *C. albicans* for glycolysis and the tricarboxylic acid (TCA) cycle. The dysregulation of the TCA cycle attenuates the production of ATP, leading to a low intracellular levels of cAMP, which dramatically affects the activation of the Ras1/cAMP-PKA pathway (Tao et al., 2017), supporting our previous results that suggested the modulatory effect of SH1009 on the ATP-binding cassette (ABC) efflux pumps which drive the fluconazole resistance mechanism (Alqahtani et al., 2019). Significantly, the top down-regulated gene in the SH1009 transcriptomic profile (by −32-fold), *HSP21*, lies downstream of the Ras1/cAMP-PKA pathway (Gong et al., 2017). The deletion of this small-heat shock protein, Hsp21, weakens *C. albicans* resistance to oxidative and thermal stresses by accumulating significantly lower intracellular levels of trehalose sugar which is considered an oxidative stress protectant (Mayer et al., 2012). The involvement of Hsp21 in the regulation of trehalose homeostasis and the tight control of trehalose level by G protein receptor (Gpr1) suggest that Hsp21 is a possible link between the Ras1/cAMP-PKA pathway and trehalose biosynthesis (Serneels et al., 2012). Additionally, SH1009 down-regulates the basic carbon metabolism that controls trehalose biosynthesis, the key nutritional and protectant cue for carbohydrate regulation and oxidative resistance in *C. albicans*.

*Candida albicans* cells exhibited a different repressed transcriptomic profile in response to SH9051 (Figure 4B). Organic sulfate compounds may be transported into the cells by the putative sulfate transporter (Seo1) for which expression was down-regulated by −2.06-fold. Sulfate, then, should be reduced through the sulfate assimilation pathway genes, *MET3*, *MET14*, *MET16*, *ECM17* (or *MET10*), which were among the top down-regulated genes (by −10, −11, −12, and −1.7-fold, respectively), to sulfide which would afterward be incorporated into the formation of homocysteine by down-regulated gene *MET15* (by −1.9-fold) along with O-acetylhomoserine derived from homoserine by the *MET2* gene (down-regulated by −1.7-fold). In addition, expression of genes involved in sulfur amino acid (methionine and cysteine) biosynthesis pathways were down-regulated. Homocysteine is converted to methionine by *MET6* (down-regulated by −2-fold) and to *S*-adenosylmethionine (SAM) in the methyl cycle by *SAM2*, *SAM4*, and *SAH1* (down-regulated by −1.5, −2, and −1.6-fold, respectively). *CYS1* and *CYS4*, which convert homocysteine to cysteine in the two steps of the transsulfuration process, were down-regulated by −2 and −1.9-fold, respectively. In *S. cerevisiae*, the levels of methionine, cysteine, and SAM negatively control the activity of transcription factor Met4p (expression down-regulated by −3-fold) that controls the activity of sulfur metabolism pathway by positively regulating the activity of *MET* genes (Ljungdahl and Daignan-Fornier, 2012). Shrivastava et al. (2021) have also recently confirmed Met4 as a regulator of methionine circuitry in *C. albicans*, observing up-regulation of many methionine biosynthesis genes, including the sulfur assimilation pathway and the SAM cycle. The findings in this present study indicate that SH9051 down-regulates the sulfur metabolism pathway which dysregulates the methionine and cysteine biosynthesis processes. Taken together, both repressed transcriptomic profiles of SH1009 and SH9051 demonstrated significantly and distinctively major perturbations of different metabolic pathways in *C. albicans* cells, suggesting different molecular targets.

### Cross-Transcriptome Profiling Reveals Overlapping Biological Processes

**FIGURE 5:**
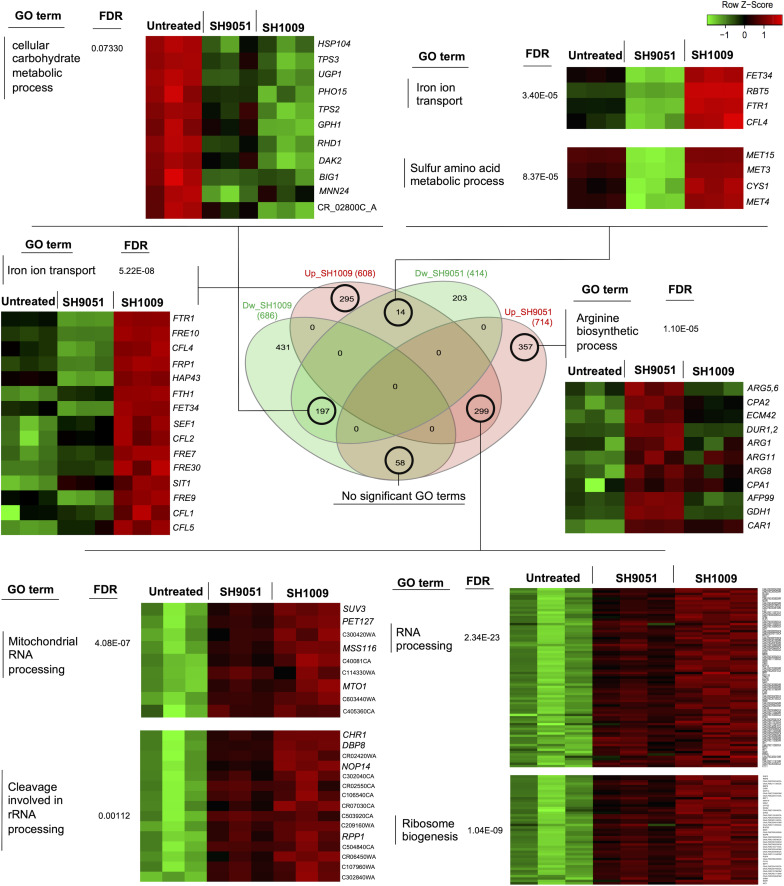
Comparisons between induced and repressed transcriptomic profiles of aurones SH1009 and SH9051. The Venn diagram shows the number of commonly or uniquely dysregulated genes between four datasets: down-regulated-SH1009, down-regulated-SH9051, up-regulated-SH1009, and up-regulated-SH9051 dataset. Heatmaps demonstrate the differential gene expression of sets of genes that are clustered and enriched for significant GO terms in each overlap. The corrected *P* values (FDR) are indicated for each GO term. The heatmaps were generated to visualize the comparisons of differential gene expression across the SH1009- and SH9051-treated and untreated samples using the shinyheatmap tool by which the expression of genes were scaled per row based on the *Z*-score of standard deviations (positive or negative) and plotted on a red–green color scale where the high level of expression is colored red and the low level of expression is colored green.

To detect any potentially synergistic or antagonistic interaction between SH1009 and SH9051 at the molecular level, a Venn intersections diagram was constructed to compare the repressed and induced transcriptomic profiles (Figure 5). Although ∼197 genes were commonly down-regulated in both repressed datasets of SH1009 and SH9051, 44% of these genes were enriched for unknown biological processes while only 11 genes were enriched insignificantly (*P* (FDR) value < 0.07) for carbohydrate metabolic processes, especially genes that are involved in drug stress response, indicating no synergistic interaction for repressed transcriptomic responses. Comparing the up-regulated genes for SH9051 and the down-regulated genes for SH1009 detected 53 commonly dysregulated genes that are not enriched for any GO term. By way of contrast, a low number of overlapped genes (only 14) for iron ion transport and sulfur amino acid metabolic processes are significantly enriched between the up-regulated genes for SH1009 and the down-regulated genes for SH9051. The up-regulated genes for sulfur amino acid metabolic processes (*MET4*, *MET3*, *MET15*, and *CYS1*) in the induced transcriptome of SH1009 could be attributed to carbohydrate starvation, which subsequently causes the cells to utilize amino acids as alternative sources of carbon (Miramón and Lorenz, 2017). The down-regulation of genes for iron ion transport (*RBT5*, *FET34*, *FTR1*, and *CFL4*) in the repressed transcriptome of SH9051-treated cells could be attributed to elevated levels of organic sulfur compounds as a consequence of sulfur amino acid catabolism. This might be concomitant with repression in iron ion import in order to hinder the synthesis of iron–sulfur (Fe-S) proteins (Petti et al., 2012). Nevertheless, this low level of antagonistically differential expression of a small number of genes cannot be interpreted as a fundamentally antagonistic interaction between the aurone modes of action because these antagonistic processes seemed to be secondary effects of the essentially targeted pathways.

However, the induced transcriptome in both SH1009 and SH9051 profiles was remarkably comparable. A large set of genes (∼299) were commonly up-regulated upon SH1009 and SH9051 treatments. These genes were significantly enriched (*P* (FDR) values < 0.0001) for RNA processing, ribosomal biogenesis, mitochondrial RNA processing, and cleavages involved in rRNA processing. High transcript levels for rRNA processing and ribosomal biogenesis in *S. cerevisiae* after exposure to high lethal concentration of H_2_O_2_ have been observed previously, suggesting an urgent need to accelerate the rRNA processing and ribosomal biogenesis that are required for synthesizing oxidative stress response proteins or to replace the rRNA that was damaged by oxidative stress (Shenton et al., 2006). Additionally, nutrient depletion has been associated with ribosomal stress by inducing ribosomal degradation and formation of free ribosomal proteins that can eventually accumulate in the nucleoplasm (Zhou et al., 2015).

In addition to commonly induced genes in response to either SH1009 or SH9051 treatments, uniquely up-regulated genes in response to each aurone have been significantly enriched for prominent biological processes. For SH1009, a set of genes was uniquely induced and enriched for iron ion transport. Most of these genes (C4_03340C_A, 11-fold; *CFL2*, 9-fold; *RBT5*, 7.9-fold; *CFL4*, 7.2-fold; *FRE9*, 4.5-fold; *FTR1*, 4.5-fold; *FTH1*, 3.6-fold; *CFL5*, 3.4-fold; *SIT1*, 3-fold; *FRE10*, 3-fold; *FRP1*, 2.8-fold; *FRE30*, 2.7-fold; C7_00430_A, 2.6-fold; *FRE7*, 2.4-fold; *FET34*, 1.9-fold; *CTR1*, 1.67-fold; and *CFL1*, 1.5-fold) were among the top up-regulated genes. The expression of iron ion acquisition genes in *C. albicans* is regulated by the induced transcriptional repressor Hap43p (two-fold), which responds to low intracellular levels of iron by inhibiting the transcriptional activator of iron utilization, Sfu1p, which was down-regulated by −1.86-fold. The Sfu1p transcription factor represses genes encoding iron uptake through direct inhibition of a third transcription factor Sef1p, for which expression was up-regulated three-fold, and indirect inhibition of Hap43p expression, since Sef1p regulates the activity of Hap43p (Chen et al., 2011). This strict regulation of iron acquisition and utilization reflects the significance of maintaining ordinary intracellular iron homeostasis to thereby prevent the iron toxicity that leads to severe oxidative stress. Overabundance of iron catalyzes the production of reactive oxygen species (ROS) by the Fenton reaction, resulting in oxidative damage in biomolecules (Missall et al., 2004). A recent study has reported that iron overload is the major determinant of the oxidative stress associated with rRNA cleavage and degradation through promoting stress-strand breaks in rRNA by ribosome-bound iron (Zinskie et al., 2018).

For SH9051, a set of genes that are involved in the arginine biosynthetic process was uniquely induced (*DUR1,2*, 8.4-fold; *GDH1*, 5.9-fold; *AFP99*, 3.6-fold; *ARG11*, 2-fold; *ARG8*, 2-fold; *ARG1*, 1.89-fold; *ECM42*, 1.9-fold; *CAR1*, 1.75-fold; *CAP2*, 1.65-fold; *ARG5*, 1.6-fold; and *CAP1*, 1.56-fold) in response to the aurone. The induction of the arginine biosynthetic pathway has been reported previously as a transcriptional response in *C. albicans* to oxidative stress resistance (Issi et al., 2017). Additionally, as a defense mechanism against host-phagocytosed cells, *C. albicans* induces the arginine biosynthetic pathway to resist macrophage-derived antimicrobial ROS (Jiménez-López et al., 2013). These findings indicate that both aurones could induce cellular oxidative stress; however, uniquely up-regulated mechanisms also suggest key roles for the functional groups in the chemical structure of each aurone.

By linking the transcriptional responses of aurones SH1009 and SH9051 to their core chemical structures and functional groups, three critical hypotheses can be proposed. First, the hydroxyl groups and/or methoxy group in aurone SH1009 can react with transition metal ions (iron), which produces high levels of toxic ROS that would result in rRNA cleavage and degradation along with ribosome stress, resulting in a potent pro-oxidant activity. Additionally, these functional groups could uniquely affect the accumulation level of the oxidative protectant, trehalose (Gencer et al., 2018), which in turn down-regulates the Ras1/cAMP-PKA pathway and negatively impacts carbon metabolism and its regulation (Perfect et al., 2017; Figure 4A). Second, since *C. albicans* utilizes amino acids as a nitrogen source, the nitrogen atom in aurone SH9051 could interfere with the negative regulation of transcription factor Met4p, simulating a high level of methionine and cysteine supply resulting in inactivation of MET genes, thus suppressing the sulfate assimilation pathway (Hébert et al., 2013) and inducing sulfur amino acid catabolism (Figure 4B). Third, the C ring structure of both aurones could contribute to generating more ROS, resulting in the common up-regulation processes (rRNA processing and ribosomal biogenesis). This would provide SH9051 a mild pro-oxidant activity, but it is lower than that of SH1009 which can be observed from the depth of the red color of the heatmap in the SH1009 gene expression profile compared to that for SH9051 (Figure 5). Therefore, a reverse genetics approach and quantitative biochemical assays were applied to validate the transcriptional signatures of aurones SH1009 and SH9051 and to establish the structure-activity relationship.

### Aurones SH1009 and SH9051 Inhibit Distinctive Carbon and Sulfur Amino Acid Metabolic Pathways

**FIGURE 6:**
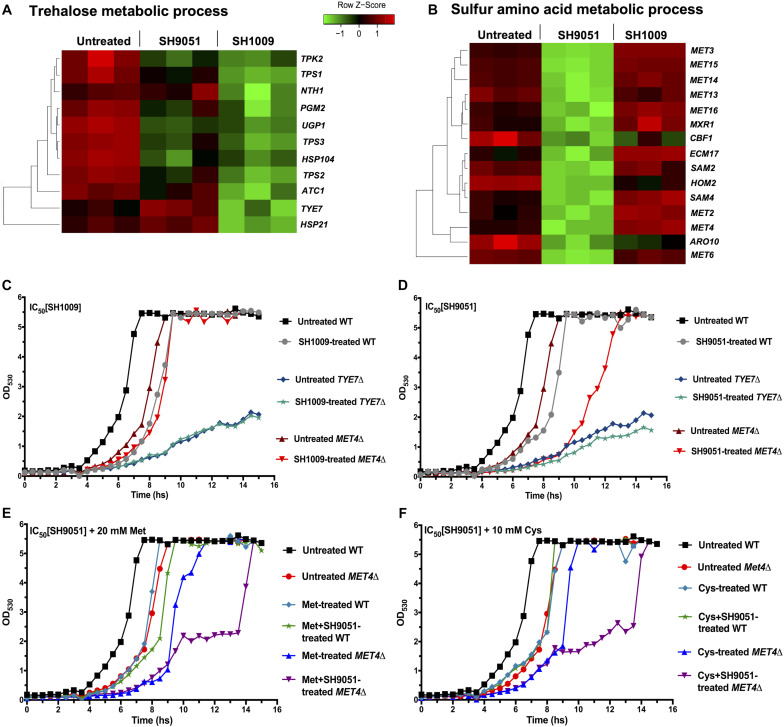
The most repressed transcriptional responses of aurones SH1009 and SH9051 treatments. **(A,B)** Aurone SH1009 and SH9051 treatments resulted in down-regulation of trehalose and sulfur amino acid metabolic processes. The relative expression of the genes of these processes was illustrated by green–red color scale in heatmap. **(C,D)** The growth curves of *C. albicans* SC5314 wild-type strain, *TYE7*Δ, and *MET4*Δ mutants under the IC_50_ concentrations of aurone SH1009 (16 μM) and SH9051 (90 μM), respectively. **(E,F)** The growth curves of *C. albicans* SC5314 wild-type strain and the *MET4*Δ mutant under the IC_50_ concentration of aurone SH9051 (90 μM) with supplements of 20 mM methionine and 10 mM cysteine, respectively. The growth conditions were in YPD broth media at 30°C using the Bioscreen C growth curve instrument with OD_530_ reads recorded at 30 min intervals for 24 h incubation.

The enrichment analysis of differentially regulated genes upon aurone treatment indicated carbon and sulfur metabolism as the most significantly down-regulated pathways. Accordingly, engineering of *C. albicans* deletion strains was attempted using the CRISPR-Cas9 gene editing technique in order to validate that the trehalose and sulfur amino acid biosynthetic metabolic pathways are the primary targets of aurones SH1009 and SH9051, respectively (Figures 6A,B). We reasoned that if the transcriptional factors of carbon metabolism, Tye7p, and sulfur metabolism, Met4p, are absent, then the aurones would not be active against the mutants. Supplementary Figure 3A depicts the genetic modification of a *TYE7*Δ mutant from a *C. albicans* SC5314 strain background by introducing double-strand breaks in which two gRNAs guide Cas9 nuclease to catalyze the cleavages at the 5′ and 3′ ends of the open reading frame (ORF) of the targeted gene *TYE7* and replace the gene sequence with the gene drive cassette (Halder et al., 2019). Although the gene drive system harbors two single gRNA sequences and SNR52 RNA polymerase III promoter, allowing the gene drive to transcript and propagate to any wild-type locus for deletion, the platform failed to generate a *MET4*Δ mutant even with multiple rounds of transformations using three different gene drive systems containing three different dual gRNAs which target different sites on the *MET4* gene sequence. Therefore, a *MET4*Δ deletion mutant was obtained from the Fungal Genetics Stock Center (FGSC) (University of Missouri, Kansas City, MO, United States), and the absence of the *MET4* gene sequence was verified by colony PCR (Supplementary Figure 3B).

The *TYE7*Δ mutant was documented previously for higher storage carbohydrate levels (trehalose and glycogen) as a result of severely reduced glycolytic flux due to the repressed expression of the genes encoding the glycolytic-committing enzyme phosphofructokinase (*PFK1* and *PFK2*). This gives Tye7p the role to regulate the flux between carbohydrate storage and production at the glucose-6-phosphate branch point in order to avoid futile glycolysis cycling (Figure 4A; Askew et al., 2009). Consequently, while the growth of *C. albicans* SC5314 was inhibited by ∼4-fold during the exponential phase of the growth curve under the IC_50_ concentration of SH1009 (16 μM), the *TYE7*Δ mutant showed evident SH1009-resistant growth (Figure 6C), suggesting that the greater storage of oxidative stress protectant (trehalose) could rescue *TYE7*Δ from the proposed pro-oxidant activity of SH1009. Additionally, it has been reported that exposing *C. albicans* to ∼30 μM extracellular iron led to accumulation of more ROS, which could cause a synergistically inhibitory activity with SH1009 (Kaba et al., 2013). However, supplying growth media with different concentrations of extracellular iron did not increase the inhibitory activity of SH1009 (Supplementary Figures 4A–C). In fact, it marginally reversed the inhibitory activity which could support the claim that the uniquely up-regulated genes for iron ion uptake signifies the low intracellular level of iron ions upon SH1009 treatment. Because *C. albicans* possess a sophisticated system of regulation of iron ion transport due to the potential toxicity of iron by generating ROS, it could be speculated that the impact of SH1009 on the level of iron ions was endogenous and it cannot be affected exogenously.

Treatment of the *MET41* mutant with SH9051 resulted in a more susceptible response compared to the wild type indicating a potential secondary effect or pleiotropic effects of the mutation (Figure 6D). However, unlike the recent findings of methionine auxotrophic phenotype in *C. albicans MET4*Δ (Shrivastava et al., 2021), our *C. albicans MET4*Δ strain is not auxotrophic for sulfur amino acids (methionine or cysteine) (Supplementary Figure 4D) which could be attributed to different genetic backgrounds. Also, the additional supplements of cysteine or methionine were not able to restore the fitness of *MET4*Δ without any stress. In addition to the SH9051 stress, addition of sulfur amino acids further inhibited the growth of *MET4*Δ (Figures 6E,F). This could support the hypothesis from RNA-seq data where SH9051 treatment stimulated a toxic supply of sulfur amino acids, suggesting a synergistically toxic effect between the SH9051 and these amino acids through sulfur amino acid catabolism. For this reason, most of the methionine and cysteine taken up by *MET4*Δ cells might be degraded thus reducing SH9051 toxicity even in the lack of *de novo* biosynthesis of these amino acids. Therefore, in order to further investigate the cellular responses of the wild type and relevant mutants during aurone SH1009 and SH9051 treatments, quantitative biochemical assays were conducted.

### Biochemical Assays Specify the Structure-Activity Relationship of Each Aurone

**FIGURE 7:**
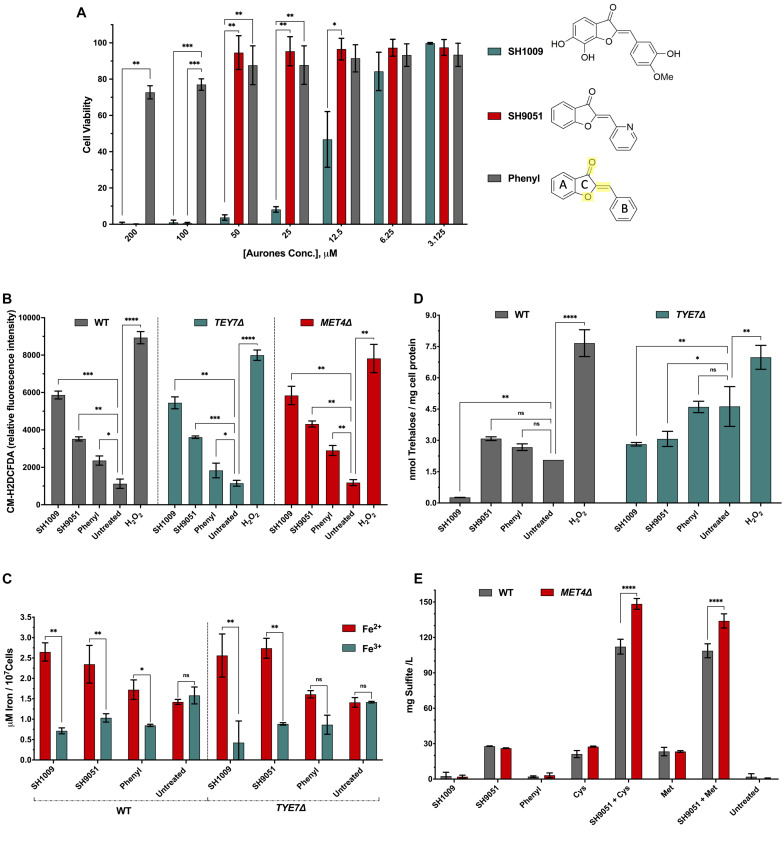
*Candida albicans* cellular growth and metabolism responses to SH1009, SH9051, and phenyl aurones. **(A)** The inhibitory activity of SH1009, SH9051, and phenyl aurones on *C. albicans* SC5314 cell suspensions (0.5–2.5 × 10^3^ CFU/mL) with treatments of two-fold serial dilutions of each aurone (3.125–200 μM) in RPMI-1640 medium for 24 h at 35°C. PrestoBlue reagent was used to assay the cell viability. C ring structure is highlighted in yellow color in the phenyl aurone where the benzofuranone heterocyclic A and C rings are connected to the phenyl B ring by extended conjugation. **(B)** Changes in ROS production after 1 h treatment of 200 μM of each aurone (H_2_O_2_ was used as a positive control) on exponentially growing *C. albicans* SC5314, *TYE7*Δ, and *MET4*Δ cells in YPD media using the fluorescent dye CM-H_2_DCFDA. **(C)** Determination of intracellular ferrous (Fe^2+^) ions in *C. albicans* SC5314 and *TYE7*Δ mutant cells based on the formation of a complex between Fe^2+^ and an iron chromogenic substrate; ferric (Fe^3+^) ions were determined using an iron reducer on separate samples to determine the total iron content and then subtracting the Fe^2+^ ions from total iron to determine the Fe^3+^ ion concentration. **(D)** The intracellular trehalose content of wild type *C. albicans* SC5314 and *TYE7*Δ at logarithmic phases were measured enzymatically following aurone treatment using trehalase coupled with Amplex red reagent as described in the “Materials and Methods” section. **(E)** Total sulfite production was measured in wild type *C. albicans* SC5314 and *MET4*Δ based on the reaction with Ellman’s reagent after treating cells with aurones and amino acids, 20 mM methionine or 10 mM cysteine. All data points are mean ± *SD* (*n* = 3). Significances were calculated using two-way ANOVA for multiple comparisons. The asterisks indicate the difference was significant. *P* values: (^****^P ≤ 0.0001), (^∗∗∗^*P* > 0.0005), (^∗∗^*P* ≤ 0.001), (^∗^*P* ≤ 0.01).

Comparisons between the repressed and induced transcriptomic profiles of *C. albicans* cells treated with aurones SH1009 and SH9051 indicated that a common feature in the aurone chemical structures could contribute to elevating intracellular ROS levels. Additionally, distinct functional groups in each aurone indicated uniquely up-regulated and down-regulated pathways (Figure 5). Accordingly, the core chemical structure of an aurone without any functional group (phenyl aurone, Figure 7A) was included in cellular metabolism assays to quantitively measure the structure-activity relationships of the aurone core and functional groups. The cell viability assay for the phenyl aurone showed that inhibition of *C. albicans* SC5314 at the highest concentration of 200 μM was approximately 28%, suggesting that approximately one third of any aurone inhibitory activity was proportionally derived from this basic chemical structure (Figure 7A).

Since the core chemical structure was the only common feature between aurones SH1009 and SH9051, we hypothesized that the C ring structures, comprised of the oxygen double bound, oxygen atom, and/or the extended conjugation that connects the benzofuranone heterocyclic ring with the phenyl group (Figure 7A), could contribute to generating ROS. Therefore, the exponential growth of *C. albicans* SC5314 wild-type, *TYE7*Δ, and *MET4*Δ cells was perturbed by 200 μM of each aurone for 1 h, after which yeast cells were stained with the oxidative stress indicator, CM-H_2_DCFDA, that is oxidized by ROS to a fluorescent molecule (Hassani and Dupuy, 2013). Intracellular ROS accumulation relative to fluorescence intensity illustrated that the highest oxidative stress was caused by SH1009 with a 77.4% significant increase (*P* value < 0.001) in ROS levels compared to the untreated sample, confirming the pro-oxidant activity of this aurone against *C. albicans* cells. In comparison, the pro-oxidant activity of the phenyl aurone raised the ROS level significantly by 45.4% (*P* value < 0.01) relative to the untreated sample. It was statistically significant that the basic structure was responsible for 24.2 and 48.1% of ROS generation in SH1009 and SH9051, respectively, relative to untreated sample (Figure 7B).

Because aurone SH1009 uniquely induced expression of iron ion transport pathways, it was speculated that treated cells would have low intracellular iron ion levels due to the catalyzing of these stress ions by the SH1009 structure, promoting the pro-oxidant activity. There are two intracellular states of transition metal iron, the oxidized form of iron, ferric (Fe^3+^) ions, which react with oxygen to form oxides with relatively low solubility, and the reduced form of iron, ferrous (Fe^2+^) ions, which react with oxygen to form ROS through Fenton reactions (Fourie et al., 2018). Typically, the reduction of ferric iron(III) to ferrous iron(II) by reductases increases the availability and solubility of iron. However, compromising the efficiency of the network controlling iron homeostasis or changing the availability of ferrous ions are highly related to toxic ROS production (Gammella et al., 2016). Therefore, the levels of Fe^3+^ and Fe^2+^ iron ions were determined using an iron probe assay (iron chromogen) after treating *C. albicans* wild type and *TYE7*Δ cells with aurones.

Figure 7C depicts the concentrations of Fe^3+^ and Fe^2+^ in treated and untreated cells where the ratio of Fe^2+^/Fe^3+^ in wild type *C. albicans* SC5314 cells treated with SH1009 was the highest (3.7) compared to treatments with SH9051 (2.2) and phenyl aurone (2.03) in wild type cells. This validates the transcriptional signature of SH1009 and supports the proposed pro-oxidant activity and the results of the ROS generation assay. Although the Fe^2+^/Fe^3+^ ratio of SH1009 in *TYE7*Δ mutant was 6.0, this strongly underscores the role of trehalose in protecting the cells since the *TYE7*Δ mutant showed an intrinsically resistant phenotype against SH1009 (Figure 6C). SH9051 treatment resulted in a 2.2 higher Fe^2+^/Fe^3+^ ratio in the wild type compared with a ratio of 2.03 in response to the phenyl aurone, suggesting that most of the ferric ion reduction trait in SH9051 (∼89.3%) could be the impact of the C ring structure. This impact contributes to (∼54%) the ability of SH1009 in catalyzing the reaction with metal ion stress (iron) which poses a question about the role of the hydroxyl groups and/or methoxy group in the trehalose level.

Intracellular trehalose levels were determined enzymatically based on hydrolysis of trehalose by trehalase into two glucose monomers after eliminating the original glucose from the samples. The glucose concentration, which is directly proportional to the trehalose concentration, was detected fluorometrically by coupling the oxidation of glucose to peroxidation of the fluorogenic substrate, Amplex Red (Carillo et al., 2013). This method overcomes the previous limitation of colorimetric detection which did not have the sensitivity to detect the low trehalose levels in exponentially growing yeast cells (Askew et al., 2009). We observed significantly increased levels of trehalose in the wild type and *TYE7*Δ cells under all treatments except the SH1009-treated wild type sample (Figure 7D). As expected and documented previously (Askew et al., 2009), the trehalose level was two times higher (*P* < 0.001) in untreated *TYE7*Δ mutant cells compared to untreated wild type cells and provides the *TYE7*Δ cells protection from the SH1009-pro-oxidant effect. SH1009 treatment considerably decreased the trehalose level in wild type cells 7.7 times (*P* < 0.001) relative to the untreated-wild type sample and 10.6 times (*P* < 0.0005) relative to the SH1009-treated *TYE7*Δ sample, demonstrating SH1009 resistance via higher trehalose storage in the *TYE7*Δ mutant. Additionally, a significant observation was the failure of the phenyl aurone to suppress trehalose synthesis with insignificant differences in trehalose concentrations compared with untreated samples either in wild type or mutant cells. This suggests that suppression of trehalose synthesis could be almost entirely attributed to the functional groups in SH1009 aurone, hydroxyl groups and/or methoxy group.

The impact of the aurone SH9051 nitrogen functional group was not only inactivation of sulfur amino acid biosynthesis, but also activation of degradation of these amino acids, simulating a high exogenous supply of methionine or cysteine (Hennicke et al., 2013; Xu et al., 2018; Figure 4B). According to previous studies, the end products of methionine catabolism are volatile organic sulfur-containing compounds (VOSCs) such as thioesters, thioethers, dimethyl trisulfide (DMTS), and dimethyl disulfide (DMDS) (Landaud et al., 2008). In *C. albicans*, large quantities of sulfite have been reported as a consequence of cysteine catabolism (Hennicke et al., 2013). To determine the role of the aurones in sulfur amino acid catabolism, total sulfite was measured based on the sulfite-triggered cleavage of the disulfide bond in Ellman’s reagent, which also reacts with compounds containing free thiols, such as VOSCs (Coulomb et al., 2017). SH9051 was the only aurone treatment that significantly (*P* < 0.0001) elevated the sulfite levels (Figure 7E) while the values produced by SH1009 and phenyl aurone treatment of cells were nearly zero, similar to untreated cells. The overproduction of sulfite associated with SH9051 treatment demonstrated the impact of the nitrogen atom on uniquely transcriptional responses to SH9051. In addition, combining SH9051 with methionine or cysteine demonstrated their synergistic effects on *C. albicans* growth (Figures 6E,F) by considerable overproduction of sulfite and VOSCs which were more significantly produced (*P* < 0.0001) in *MET4*Δ mutant than wild type cells, demonstrating the hypersensitivity of *MET4*Δ toward SH9051 (Figure 6D). These observations indicate that Met4p activity in *C. albicans* might not only be involved in sulfur metabolism but also participates in defense against several stresses, including oxidative stresses.

## Discussion

The aim of our study was to comprehensively understand the modes of action for aurones SH1009 and SH9051 treatment in *C. albicans* SC5314 using the global transcriptional signature and leveraging the power of the RNA-seq technique. Gene expression analysis showed that aurones SH1009 and SH9051 led to the dysregulation of a staggering number of diverse genes (1249 and 1085 genes, ∼21 and 19% of the genome, respectively for SH1009 and SH9051). These genes are primarily enriched for different metabolic pathways, specifically carbohydrate and sulfur metabolisms in response to SH1009 and SH9051, respectively. The pathogenicity of *C. albicans* is linked to metabolic processes to support the bioenergetic requirements of infectious growth. Thereby, one of the most important metabolic contributors to the pathogenicity of this opportunistic fungus is stress adaptation by synthesizing the disaccharide, trehalose (Brown et al., 2014).

The transcriptional response to aurone SH1009 treatment showed that trehalose metabolism was the most significantly repressed biological process. This non-reducing sugar serves as not only carbohydrate storage in fungal cells, but also accumulates in response to thermal and oxidative stresses. Due to its physical interaction with proteins and phospholipids as a highly hydrophilic molecule, trehalose plays a critical role in stabilizing enzyme activities and protecting cellular membranes during stress. Trehalose acts as a free radical scavenger under ROS-induced conditions, supporting the fungal pathogenicity against the human immune system defense by accumulating this stress protectant through up-regulation of trehalose biosynthesis genes (Missall et al., 2004). Trehalose biosynthesis and storage are tightly linked to the glycolytic pathway and controlled by transcriptional regulator Tye7p (Askew et al., 2009). The uniquely repressed effect of aurone SH1009 on the transcription regulator Tye7p, as evidenced by RNA-seq data, showed by far the greatest impact as a regulator of 44% of down-regulated genes. The result of deleting this transcription factor (*TYE7*Δ) was an SH1009 resistant mutant (Figure 6C) that uses the higher storage of oxidative stress protectant (trehalose) as an intrinsic line of defense (Figure 7D). Previous studies have shown that disruption of the trehalose biosynthesis pathway results in dysregulation of the central carbon metabolism and alteration in stress response and virulence. In *C. albicans*, disruption of Tps1 or Tps2 increased the susceptibility to oxidative stress and attenuation of virulence in a systemic mouse model (Perfect et al., 2017; Thammahong et al., 2017). Furthermore, in trehalose-deficient mutants, the enzymatic antioxidant activities were synergistically induced during exposure to oxidative stress inducer H_2_O_2_ (González-Párraga et al., 2003).

Typically, treating *C. albicans* with H_2_O_2_ leads to increased trehalose content and up-regulation of enzymatic antioxidant activities that are considered oxidative stress defenses (Pedreño et al., 2006). These defenses include a single catalase gene for conversion of H_2_O_2_ to water and oxygen (Wysong et al., 1998), six superoxide dismutase SOD genes that convert superoxide anions to hydrogen peroxide subsequently processed by catalase (Hwang et al., 2003), four glutaredoxins GRX genes (Chaves et al., 2007), and two thioredoxin TRX genes, all of which are involved in the oxidative stress response (Dantas et al., 2010). However, none of these antioxidant enzymes were differentially expressed upon SH1009 treatment. Nonetheless, these antioxidant enzymes were modulated by the pro-oxidant activity of SH1009 because, in *C. albicans*, these enzymes are regulated by iron availability (Chakravarti et al., 2017). SH1009 uniquely up-regulated Hap43p, which has been recorded as the sole transcriptional regulator responsible for iron-dependent oxidative stress response. Under iron deprivation conditions, it acts as a transcriptional activator for iron ion uptake genes (Hsu et al., 2011) and as a repressor of numerous genes encoding enzymes that utilize iron as a cofactor, including antioxidant enzymes (Cat1, Sod4, Grx5, and Trx1), possibly to permit the utilization of iron in more essential processes (Chakravarti et al., 2017).

The pro-oxidant activity has been identified as the ability to reduce transition metal ions ferric(III) to lower oxidation states ferrous(II) that increase the production of ROS (Truong et al., 2020), which can be used in cell signaling depending on context and concentration. However, ROS are highly reactive molecules and can inflict damage to cellular macromolecules (Shcherbik and Pestov, 2019) like cleavages involved in rRNA and ribosomes by stress-strand breaks via ribosome-bound iron (Zinskie et al., 2018), which was the commonly up-regulated process for both SH1009 and SH9051 treatments. The structure-activity relationship metabolic assays evidenced the role of the C ring structure in generating ROS through disruption of iron homeostasis. Based on the phenyl aurone treatments, the core chemical structure contributes to 24.2% of SH1009 activity for generation of ROS and 51.3% for catalyzing iron ions. Our results support prior studies that suggested the role of oxygen atoms and the double bond of the C ring structure in increasing pro-oxidant potential in flavonoids. Also, the number of hydroxyl groups, their positions, and methoxylated B ring were important factors for the structure-pro-oxidant activity (Eghbaliferiz and Iranshahi, 2016; Baldim et al., 2017). These functional groups complete the residual pro-oxidant potency of SH1009 by contributing 75.8% to ROS generation, 48.7% to catalyzing iron ions, and most significantly, 100% of the impact on trehalose protection in *C. albicans*.

Although flavonoid compounds are naturally synthesized as antioxidant metabolites in plants, the pro-oxidative properties of these compounds can be enhanced in the presence of iron(III) or copper(II) ions mainly because of their ability to reduce metal ions based on Fenton-type reactions and generate ROS (Eghbaliferiz and Iranshahi, 2016). This property allows flavonoids to selectively target particular cells with characteristics such as extra quantities of redox metal ions. For example, it has been observed that flavonoids can boost cellular levels of ROS to cytotoxic ranges in cancer cells but not in normal cells due to the higher concentration of copper(II) ions in the metabolically active cancer cells (Eghbaliferiz and Iranshahi, 2016; Jomová et al., 2019). In this regard and supporting our previous work showing the selective toxicity of SH1009 for *C. albicans* over human cells (Alqahtani et al., 2019), the selectively pro-oxidant potency of SH1009 could be attributed to the naturally high redox metal iron ions inside *C. albicans* cells. Whereas iron ions in the host are largely unavailable as they are constantly sequestered by iron-binding proteins like heme and ferritin, *C. albicans*, as a commensal opportunistic pathogen, has evolved sophisticated iron acquisition and storage systems which enable this fungus to scavenge iron from the host and store it in compartments such as vacuoles and mitochondria (Fourie et al., 2018). The pathway that controls the intracellular iron mobilization and storage has been identified in *C. albicans*. The two vacuolar transporters Ccc1 and Smf3 were dysregulated upon SH1009 exposure where Ccc1 (expression up-regulated by 2.14-fold) increases vacuolar storage of iron and Smf3 (expression down-regulated by −1.8-fold) appears to play a role in vacuolar export (Xu et al., 2014). Seemingly, *C. albicans*, under SH1009 exposure, conceals free iron ions from the cytoplasm by storage in vacuoles as a detoxification mechanism to avoid the iron(III)-SH1009 reduction effect. Furthermore, the genes, *CCC1* and *SMF3*, are controlled by the up-regulated Hap43p, indicating that regulation of iron ion uptake was not only for acquiring iron from external sources but also to control intracellular iron homeostasis (Singh et al., 2011; Monteiro et al., 2020).

The structure-activity relationship also indicated that aurone SH9051 induces a mild pro-oxidant activity by generating ROS through catalyzing iron ions. Even though SH9051 reduction of metal ions was primarily (86.3%) derived from the C ring structure, SH9051 generation of ROS was much higher than the phenyl aurone (51.4%), indicating that another factor increases ROS generation with SH9051 treatment. It has been shown that growth inhibition of *C. albicans* occurs after addition of sulfite as reactive sulfur species (RSS), suggesting an increase in the intracellular oxidative stress (Ogasawara et al., 2008). Previous work on *S. cerevisiae* also documented induction of ROS levels in volatile sulfur compound (SO_2_)-challenged cells (Lage et al., 2019). This leads us to conclude that the increased effects of ROS generation in SH9051 compared to the phenyl aurone were due to higher accumulation of sulfite and VOSCs as byproducts of sulfur amino acid catabolism, which is essentially the unique impact of the nitrogen atom in SH9051.

Although methionine catabolism has not yet been intensively studied in *C. albicans*, based on previous studies in the industrial yeasts *Kluyveromyces lactis* (Kagkli et al., 2006), *Yarrowia lipolytica* (Hébert et al., 2013), and the mycoparasitic fungus *Clonostachys rosea* (Xu et al., 2018), methionine can be degraded cellularly through Ehrlich and demethiolation pathways into VOSCs by different steps (Kagkli et al., 2006). Even with poor enrichment of the genes that encode the enzymes of the Ehrlich pathway in *C. albicans*, essential genes in this pathway were detected in the RNA-seq data, including the up-regulated aminotransferases (ARO8, three-fold and ARO9, 2.4-fold) and oxidoreductases (ADH5, two-fold and ADH4, 102.3-fold) (Figure 4B). Cysteine catabolism was investigated previously in *C. albicans* in which the cells responded to high levels of cysteine by activation of the transcription factor Zcf2p (expression up-regulated 3.46-fold) which regulates the cysteine dioxygenase Cdg1 (expression up-regulated 3.5-fold) to convert cysteine to sulfite. As a result, the toxic accumulation of sulfite is exported via the highly up-regulated sulfite flux pump, Ssu1 (expression up-regulated 32-fold) (Figure 4B; Hennicke et al., 2013).

Sulfur amino acid biosynthesis and catabolism are regulated by Met4p, which is naturally inactivated by excess levels of methionine and cysteine. The *MET4*Δ mutant was inhibited by SH9051 treatment even with methionine and cysteine additions (Figures 6D–F), indicating that the SH9051 caused the same effects as excess levels of these amino acids. Because methionine intracellularly shuttles organic nitrogen and the amino acids play a key role in intracellular nitrogen availability in eukaryotic cells (Pratelli and Pilot, 2014), SH9051, as a nitrogenous compound, could increase intracellular nitrogen overabundance. The up-regulation of nitrogen catabolite repression pathway genes, which control the detoxification of poor nitrogen supply, *GAT1*, *GZF3*, and *UGA3* (up-regulated by 1. 77-, 2. 02-, and 1.52-fold, respectively) was detected. The activation of this pathway has also been reported as a response to high-toxic levels of ammonium in *S. cerevisiae* by increasing amino acid excretion (Hess et al., 2006). In *C. rosea*, the activation of the Ehrlich pathway and demethiolation in response to highly exogenous methionine was regulated by nitrogen catabolite repression (Xu et al., 2018). For *C. albicans*, in addition to up-regulation of the nitrogen catabolite repression pathway that is associated with sulfur amino acid catabolism (Chebaro et al., 2017), the arginine biosynthesis pathway has been linked to the urea cycle (Jastrzębowska and Gabriel, 2015). Under poor nitrogen supply, the arginine biosynthesis pathway is induced in order to regulate the urea amidolyase, Dur1,2 (expression up-regulated by nine-fold), which converts excess nitrogen to CO_2_ and ammonia in order to export the latter out of the cell through the up-regulated ammonia transport pumps (*DUR3* 1.8-fold, *MEP1* 3-fold, *ATO5* 4.7-fold, *TOP2* 1.8-fold, *TPO4* 1.6-fold, *FLU1* 2.6-fold, and *RTA2* 6.8-fold) (Vylkova et al., 2011).

In this study, the RNA-seq technique comprehensively presented the molecular mechanisms behind the inhibitory activity of aurones SH1009 and SH9051 against *C. albicans* cells and interpreted the selectivity of SH1009 against fungal cells. The diminished trehalose levels combined with elevated intracellular ROS content synergistically contribute to cell damage, ultimately affecting RNA processing and ribosome biogenesis, which could be a promising therapeutic strategy by making *C. albicans* more vulnerable to the host immune oxidative burst. Targeting the trehalose metabolic biosynthesis pathway has been identified as an attractive target for antifungal drug development, not only because of the important role of trehalose in progressing the *Candida* infection (Perfect et al., 2017), but also because of the absence of any functional trehalose synthase gene in vertebrates (Argüelles, 2017), which makes SH1009 relatively non-toxic for human cells (Alqahtani et al., 2019). The sulfate assimilation pathway has also been recognized as a favorable antifungal target since humans cannot synthesize methionine like yeasts (Jastrzębowska and Gabriel, 2015). However, the nitrogen atom in SH9051 could increase urea levels in human cells as a nitrogenous waste product because, in mammals, excess nitrogen is excreted as urea (Wright, 1995), which could explain the low selectivity index of SH9051. The paralleled analysis of the transcriptional responses of the aurone SH1009 and SH9051 simultaneously allowed us to examine the overlapping enrichment terms between the repressed and induced transcriptomic profiles, potentially clarifying the antifungal roles for the C ring structure and functional groups. The knowledge gathered in this work can be used for development and design of more efficient aurone compounds with highly selective toxicity and pave the way for a better understanding of how the molecular mechanisms exerted by these compounds can be exploited for a therapeutic approach.

## Materials and Methods

### Materials and Reagents

*Candida albicans* SC5314 strain was purchased from the American Type Culture Collection (Manassas, VA, United States). The *C. albicans MET4*Δ mutant (SFY39) was prepared by Aaron Mitchell and obtained from the FGSC (University of Missouri, Kansas City, MO, United States). YPD agar and broth, 3-(N-morpholino) propanesulfonic acid (MOPS) buffer, dimethyl sulfoxide (DMSO), BeadBug^TM^ pre-filled tubes, dithiothreitol (DTT), nourseothricin sulfate (NAT), and ampicillin (AMP) were all purchased form MilliporeSigma (St. Louis, MO, United States). RPMI-1640 medium was purchased from Corning Incorporated (Corning, NY, United States). PrestoBlue, TRIzol^TM^ Reagent, phosphate buffered saline (PBS), salmon sperm DNA, lithium acetate (LiAc), polyethylene glycol (PEG) 3350, RiboPure^TM^-DNase I Treatment, CM-H2DCFDA (Oxidative Stress Indicator), and Amplex^TM^ Red Glucose/Glucose Oxidase Assay Kit were purchased from Life Technologies Corporation (Carlsbad, CA, United States). CutSmart Buffer, *Ngo*MIV, *Pac*I, Xho1 restriction enzymes, Phusion high-fidelity (HF) DNA polymerase, 2 × Gibson assembly mix, and Chemically competent DH5α *Escherichia coli* cells were purchased from New England BioLabs (Ipswich, MA, United States). The RNase-Free DNase Kit, Miniprep plasmid extraction kit, Gel extraction kit, and PCR purification kit were purchased from QIAGEN (Germantown, MD, United States). The *E. coli* bacterial culture containing the CaCAS9 plasmid (reference number 89576) was purchased from Addgene Headquarters (Watertown, MA, United States). The trehalose assay kit and total sulfite assay kit were purchased from Megazyme Inc. (Chicago, IL, United States). The iron assay kit (colorimetric) was purchased from Abcam (Cambridge, MA, United States). Drop-out mix synthetic growth medium, minus methionine and cysteine was purchased from United States Biological Life Sciences (Salem, MA, United States).

### Checkerboard Assays

Stock solutions of aurone SH9051 and SH1009 were prepared in DMSO at a high concentration of 40 mM. Aurone interactions were carried out in RPMI 1640 medium (adjusted to pH 7.0 with 0.165 M of MOPS buffer) in 96-well flat-bottomed microtitration plates using broth microdilution checkerboard assays as described previously (Shrestha et al., 2015). Two-fold serial dilutions were initially prepared at four times the desired final concentrations (3.125–200 μM) of aurones. Aliquots of 50 μL of each concentration of SH9051 were added to columns 1–7, and then 50 μL of SH1009 was added to rows A to G. Row H and column 8 contained the two-fold serial dilutions of SH9051 and SH1009 alone, respectively. Column 9 was the drug-free well that served as negative control. The inoculum of *C. albicans* strain SC5314 was prepared as described previously (Alqahtani et al., 2019). The final concentrations of SH9051 and SH1009 after adding 100 μL of the prepared inoculum (0.5–2.5 × 10^3^ CFU/mL) ranged from 200 to 3.125 μM. The microtiter plates were incubated at 35°C for 24 h. PrestoBlue reagent was used to detect the percentage of fluorescent-viable cells in each well using a SpectraMax M5e spectrophotometer (Molecular Devices, LLC, United States). The minimum inhibitory concentration (MIC) was defined as the concentration that reduced the growth by 90%. The assay was performed in triplicate and the IC_50_ values were calculated using GraphPad Prism (GraphPad Software, United States). Drug interaction was estimated by FICI model. This model was determined according to the formula: FIC SH1009 = MIC SH1009 in combination/MIC SH1009 alone. FIC SH9051 = MIC SH9051 in combination/MIC SH9051 alone. FIC index = FIC SH1009 + FIC SH9051. FICI value was interpreted to characterize aurone compounds interactions as; FIC index ≤ 0.5 = synergy, FIC index 0.5 ≤ 1.0 = additivity, FIC index 1.0 ≤ 4.0 = indifference, FIC index > 4.0 = antagonism.

### Cytotoxicity Assay

Three human cell lines (A549 human lung carcinoma epithelial, human monocytic THP-1, and HepG2 human liver carcinoma epithelial cells) were used to assay the toxicity of aurone SH9051. Human cell lines were grown, maintained and seeded into 96-well microtiter plates at a density of 1.0 × 10^5^ viable cells/well as described previously (Alqahtani et al., 2019). After seeding, the cells were treated with two-fold serial dilutions (3.125–200 μM) of aurone SH9051. After incubation for 24 h at 37°C with 5% CO_2_ in a humidified incubator, the cell viability was evaluated using 20 μL of PrestoBlue. The assay for each cell line was performed in triplicate. The 50% cytotoxicity concentration values were calculated using GraphPad Prism (GraphPad Software, United States).

### Genome-Wide Transcriptional Analysis

#### RNA Extraction, Processing, and Sequencing

After growing *C. albicans* SC5314 cells at 30°C in YPD broth for 12 h, the culture was diluted 1:200 and allowed to grow with agitation at 30°C until reaching early exponential phase (OD_530_ = 0.15). The cell cultures (∼1–1.5 × 10^7^ CFU/mL) were treated with aurone SH1009 or SH9051 at the concentration that caused ∼30% inhibition (400 and 200 μM, respectively) followed by incubation for an additional 1 h. Solvent (DMSO) was added to the negative control culture to reach the concentration of 1%. After harvesting the cells by centrifugation, yeast cells were disrupted mechanically with BeadBug^TM^ pre-filled tubes, containing glass beads. Afterward, the total RNA were extracted according to TRIzol^TM^ reagent protocol (Rio et al., 2010) and treated with RiboPure^TM^-DNase I reagent to remove any residue of chromosomal DNA. Total RNA from nine biological-independent replicates of SH1009-treated samples (three replicates), SH9051-treated samples (three replicates) and untreated samples (three replicates) were checked for their quantities, qualities, and integrities using NanoDrop^TM^ Lite Spectrophotometer (Thermo Fisher Scientific, Waltham, MA, United States), agarose gel electrophoresis, and Bioanalyzer 2100 system (Agilent Technologies, Santa Clara, CA, United States). Volumes of ∼20 μL of high-quality total RNA of all samples were sent to Novogene Corporation Inc. (Sacramento, CA, United States) for library preparation and RNA sequencing.

#### RNA-seq Data Analysis

An average of ∼26873797 paired-end reads per sample (150 bp length) were received in FastQ files. Pre-processing quality control, mapping, quantification, and statistical summarizing of sequence reads were accomplished using web-based tools through the stand-alone platform, Galaxy server (Goecks et al., 2010). To enhance mappability, raw sequence reads were filtered by trimming low quality reads (Qscore < 20), adaptor sequences, and sequences containing N using FastQC (Leggett et al., 2013) and Trimmomatic (Bolger et al., 2014). Clean reads (∼98.55% of raw reads) were deposited at the Gene Expression Omnibus (GEO) database under the accession numbers GSE158472. The processed sequence reads were aligned to reference *C. albicans* genome SC5314_A21 using HISAT2 (Kim et al., 2015). The alignments for each sample was assembled using the *C_albicans*SC5314__A21_features.gff annotation file with StringTie (Pertea et al., 2015). The alignments with mapping quality <20 scores were excluded. After assembling all samples, assembled transcripts were merged together by StringTie-merge in order to create a uniformly new set of transcripts for all the samples and restore any missing exon in any transcript in one sample due to the possibility of lacking sufficient coverage (Pertea et al., 2016). The merged transcripts were used as an input file to quantify the transcripts in each sample using featureCounts (Liao et al., 2014). Then, the Limma-voom tool compared the transcripts level of SH1009-treated and SH9051-treated samples to the transcripts level in untreated samples (Law et al., 2014). The count data were normalized with trimmed mean of *M* value (TMM) method after filtering out the genes (unexpressed genes) with <1 count per million reads. Differentially expressed genes were determined for each treatment as ≥1.5-fold change with corrected *P* values that control the false discovery rate at 5%. The GO enrichment analysis was conducted with the Candida Genome Database GO TermFinder (Boyle et al., 2004) and FungiDB data base (Basenko et al., 2018).

### CRISPR/Cas9 Mediated Gene Deletion Strain Construction

The CRISPR/Cas9 gene editing was used to delete the *TYE7* (C2_04060C) allele based on the gene drive platform as described previously with minor modifications (Halder et al., 2019). Briefly, sequences of gene drive constructs, which contain synthetic guide RNAs (sgRNAs), repair templates, and SNR52 RNA polymerase III promoter, and their verification primers are listed in Supplementary Table 4. The CaCAS9 plasmid contains a cassette of the *Candida* codon-optimized CAS9 endonuclease gene, NAT, and ampicillin (AMP) cassettes (selective markers), gene drive cloning site, and NEUT5 locus sequences for genomic integration. After digesting the CaCas9 plasmid with *Ngo*MIV, ∼1000 bp Oligo pairs of *TYE7*-gene drive were cloned separately to CaCas9-digested plasmid using Gibson assembly master mix. Then, 1 μL of ligation reactions were introduced into chemically competent DH5α *E. coli* cells according to the manufacturer’s transformation protocol. Transformants were selected on LB agar + ampicillin. Plasmids with corrected-orientation inserts were verified by amplifying the insert region with universal primers (PAC6725_F and PAC6389_R) and with plasmid fingerprinting using Xho1. After digesting with *Pac*I to linearize the plasmids, ∼1.5 μg were transformed into *C. albicans* cells (SC5314 background) with the LiAc/ssDNA/PEG/DTT method. Fungal cells were recovered in YPD broth for 4 h and then plated on YPD agar supplemented with 250 μg/mL of NAT. After incubation for 4 days at 30°C, NAT^*R*^ transformants were plated again onto fresh YPD agar + NAT plates. NAT^*R*^ mutants were validated for their desired gene deletions using colony PCR and gel electrophoresis.

### Growth Rate Assay

The inocula of *C. albicans* SC5314 wild type, *TYE7*Δ, and *MET4*Δ cells were prepared in YPD broth media at working concentrations of 1–5 × 10^3^ CFU/mL, and 100 μL were added into the wells of a 100-well Bioscreen honeycomb plate. Then, 100 μL of SH1009 or SH9051 were added to respective wells to reach the final concentrations of the IC_50_, 16 and 90 μM, respectively, with or without the supplements of methionine, cysteine, and iron(II) chloride. Plates were placed into the Bioscreen C instrument with Bioscreen software (Growth Curves USA, Piscataway, NJ, United States) at a temperature of 30°C with continuous shaking for 24 h. The optical density reads (OD_530_) were measured at 30 min intervals to monitor the growth curves.

### Measurement of ROS Levels

*Candida albicans*, *TYE7*Δ, and *MET4*Δ cells from an overnight culture were diluted in fresh YPD to an OD_530_ of 0.12 and allowed to grow to the early exponential phase (OD_530_ = 0.15). Cells were pelleted in 96-wells plate at concentration of (2.5–5 × 10^5^ CFU/mL) and treated with 200 μM of SH1009, SH9051, and phenyl aurone and incubated for 1 h at 30°C. ROS levels were assessed using the fluorescent dye chloromethyl-dichlorodihydrofluorescein diacetate (CM-H2DCFDA) at a final concentration of 20 μM. Cells were incubated at 37°C for 1 h. The fluorescence intensity was measured with a 485 nm excitation and 535 nm emission using SpectraMax M5e spectrophotometer (Molecular Devices, LLC, United States). ROS accumulation was calculated with respect to background fluorescence.

### Intracellular Iron Content and Trehalose Determinations

Cultures of *C. albicans* strains SC5314 and *TYE7*Δ (*MET4*Δ mutant was excluded because of the limited resources) were grown until reaching the exponential phase (as descried in measurement of ROS levels experiment) and plated in 12-well plates at a concentration of 1 × 10^7^ CFU/mL and treated with 200 μM of SH1009, SH9051, or phenyl aurone, and incubated for 1 h at 30°C. After treatment, cells were washed once with water, and resuspended in water. Afterward, cells were lysed mechanically with BeadBug^TM^ pre-filled tubes, containing glass beads. A 50 μL volume of each sample was plated in a 96-well plate for intracellular ferrous iron (Fe^2+^) and ferric iron (Fe^3+^) ions determination according to the instructions of Abcam iron assay kit (colorimetric).

For trehalose determination, yeast cell lysates were prepared by incubation at 95°C for 30 min, after which 200 μL of supernatants were used for enzymatic analysis (Megazyme Trehalose Assay Kit) following the manufacturer’s procedure with modifications. Briefly, 10 μL of solution 2 (NADP+ and adenosine 5′-triphosphate), 20 μL of solution 1 (buffer), and 2 μL of suspension 3 (hexokinase and glucose-6-phosphate dehydrogenase enzymes) were added to the wells to convert the glucose to gluconate-6-phosphate. After incubation with constant shaking for 60 min at 30°C to remove glucose, the reaction plate was sealed and heated at 90°C for 15 min to inactivate the enzymes. After cooling, 2 μL of suspension 4 (trehalase enzyme) was added to wells, and the plate was incubated at room temperature for 8 min. The released glucose monomers were assayed fluorometrically using the Amplex^TM^ Red Glucose/Glucose Oxidase Assay Kit. Fluorescence readings were converted to absolute concentrations of trehalose based on an internal calibration curve of nmol trehalose standards and normalized for the cell protein concentration (mg) using the BCA protein assay.

### Detection of Sulfite

A commercially available total sulfite detection kit (Megazyme) was used for sulfite identification in *C. albicans* and *MET4*Δ cultures. In brief, cultures of strains SC5314 and *MET4*Δ were grown in YPD media (as described in the measurement of ROS levels). After reaching exponential phase, cells were treated with 200 μM of each aurone, 20 mM methionine, and 10 mM cysteine for 1 h at 30°C. Afterward, sulfite concentrations were measured according to the instructions of the manufacturer. All sample measurements were normalized to the respective background blank media.

## Data Availability Statement

The datasets presented in this study can be found in online repositories. The names of the repository/repositories and accession number(s) can be found in the article/Supplementary Material.

## Author Contributions

FA, SH, and MF designed the conceptualizations. FA analyzed and visualized the data and wrote the manuscript. MF and SH administrated and funded the project and reviewed the manuscript. MF supervised the project. All authors carried out the experiments.

## Conflict of Interest

The authors declare that the research was conducted in the absence of any commercial or financial relationships that could be construed as a potential conflict of interest.

## Supplementary Material

Supplementary Figure 1 Cytotoxicity assay of SH9051.

Supplementary Figure 2 Diagnosis plots of RNA-seq data quality.

Supplementary Figure 3 Verification of mutant genotypes.

Supplementary Figure 4 Growth curves of SH1009 + FeCl_3_ treatments.

Supplementary Table 1 Selectivity index of SH9051.

Supplementary Table 2 Gene expression analysis of SH1009.

Supplementary Table 3 Gene expression analysis of SH9051.

Supplementary Table 4 List of gene drive construct and primers.

Supplementary File 1 RNA samples quantity and integrity.
