# Supplementary material for: Combining Genome-Wide Gene Expression Analysis (RNA-seq) and a Gene Editing Platform (CRISPR-Cas9) to Uncover the Selectively Pro-oxidant Activity of Aurone Compounds Against Candida albicans
Source: Front Microbiol. 2021 Jul 15;12:708267. doi: 10.3389/fmicb.2021.708267 (PMC8319688; doi:10.3389/fmicb.2021.708267)
Supplement: Supplementary Figure 1 — Cytotoxicity assay of SH9051. [file Data_Sheet_1.pdf]

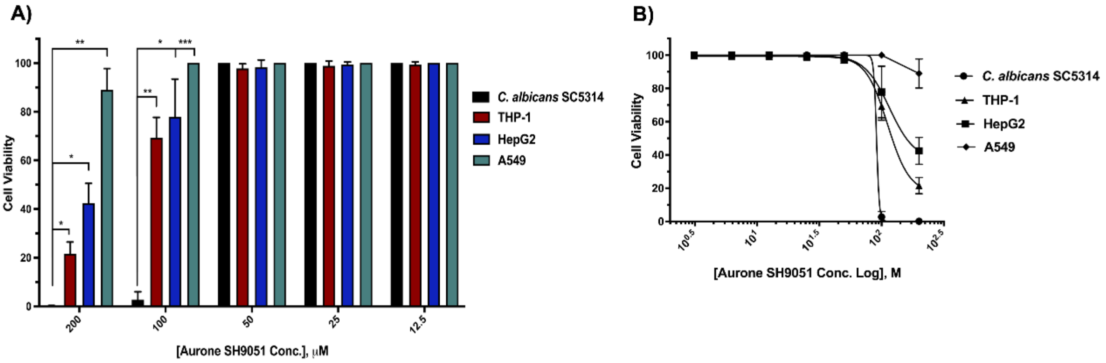

S1 Figure: The cytotoxic effects of aurone SH9051 on *C. albicans* SC5314 and human cell lines (THP-1, HepG2, and A549). **A)** Significance was calculated using two-way ANOVA to compare the cell viability of *C. albicans* to the viability of human cell lines. *P* values (*** $P \leq 0.001$), (** $P \leq 0.01$), (* $P \leq 0.05$). **B)** Dose-response curves of SH9051 (logarithmic form of molar concentrations) treatment against *C. albicans* SC5314 and human cell lines (THP-1, HepG2, and A549) by graphing the cell viability reading as nonlinear regression using GraphPad Software.
