# Supplementary material for: Combining Genome-Wide Gene Expression Analysis (RNA-seq) and a Gene Editing Platform (CRISPR-Cas9) to Uncover the Selectively Pro-oxidant Activity of Aurone Compounds Against Candida albicans
Source: Front Microbiol. 2021 Jul 15;12:708267. doi: 10.3389/fmicb.2021.708267 (PMC8319688; doi:10.3389/fmicb.2021.708267)
Supplement: Supplementary Figure 2 — Diagnosis plots of RNA-seq data quality. [file Data_Sheet_2.pdf]

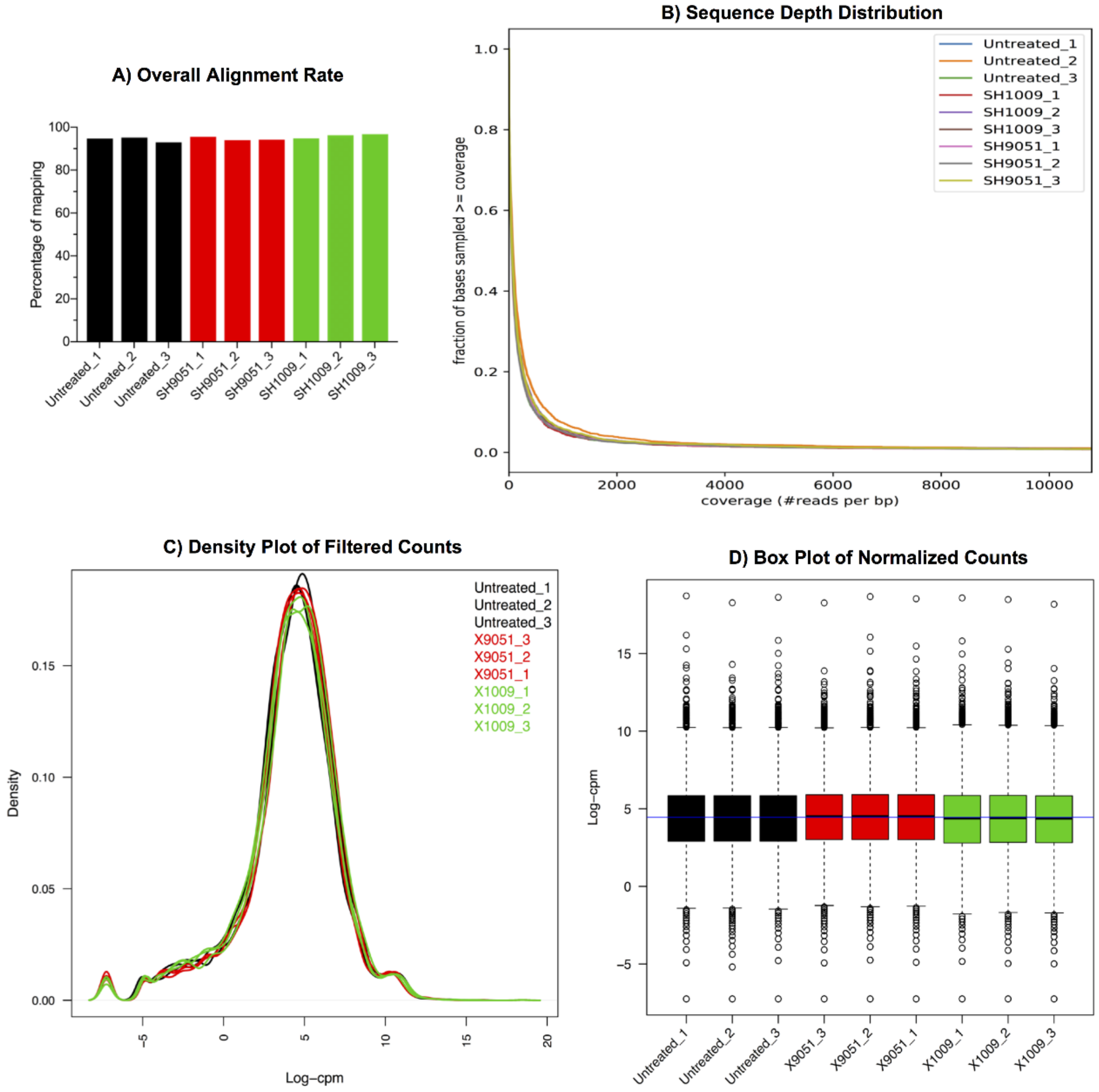

S2 Figure: Diagnostic plots for quality assessment of RNA-seq data. A) The quality assessment of mapped reads showed high alignment accuracy with an average of 94.88% mapping rate (HISAT2 tool). **B)** The distribution of fragment coverage for all of the 9 samples showed that < 10% of sampled base pairs have up to 1000 overlapping reads, indicating a uniformly sufficient sequencing depth (plotCoverage tool). **C)** The distribution of filtered counts, using a count per million (CPM) threshold of 1 to remove low-count genes, showed consistent frequency profiles for all the samples, indicating high quality of RNA purification and library preparation steps with no difference in RNA composition among the samples (Limma-voom tool). **D)** The distribution of normalized counts showed a straight blue line crosses the gene expression at the median level evenly, indicating a proper normalization method (Limma-voom tool).
