# Supplementary material for: Combining Genome-Wide Gene Expression Analysis (RNA-seq) and a Gene Editing Platform (CRISPR-Cas9) to Uncover the Selectively Pro-oxidant Activity of Aurone Compounds Against Candida albicans
Source: Front Microbiol. 2021 Jul 15;12:708267. doi: 10.3389/fmicb.2021.708267 (PMC8319688; doi:10.3389/fmicb.2021.708267)
Supplement: Supplementary Figure 3 — Verification of mutant genotypes. [file Data_Sheet_3.pdf]

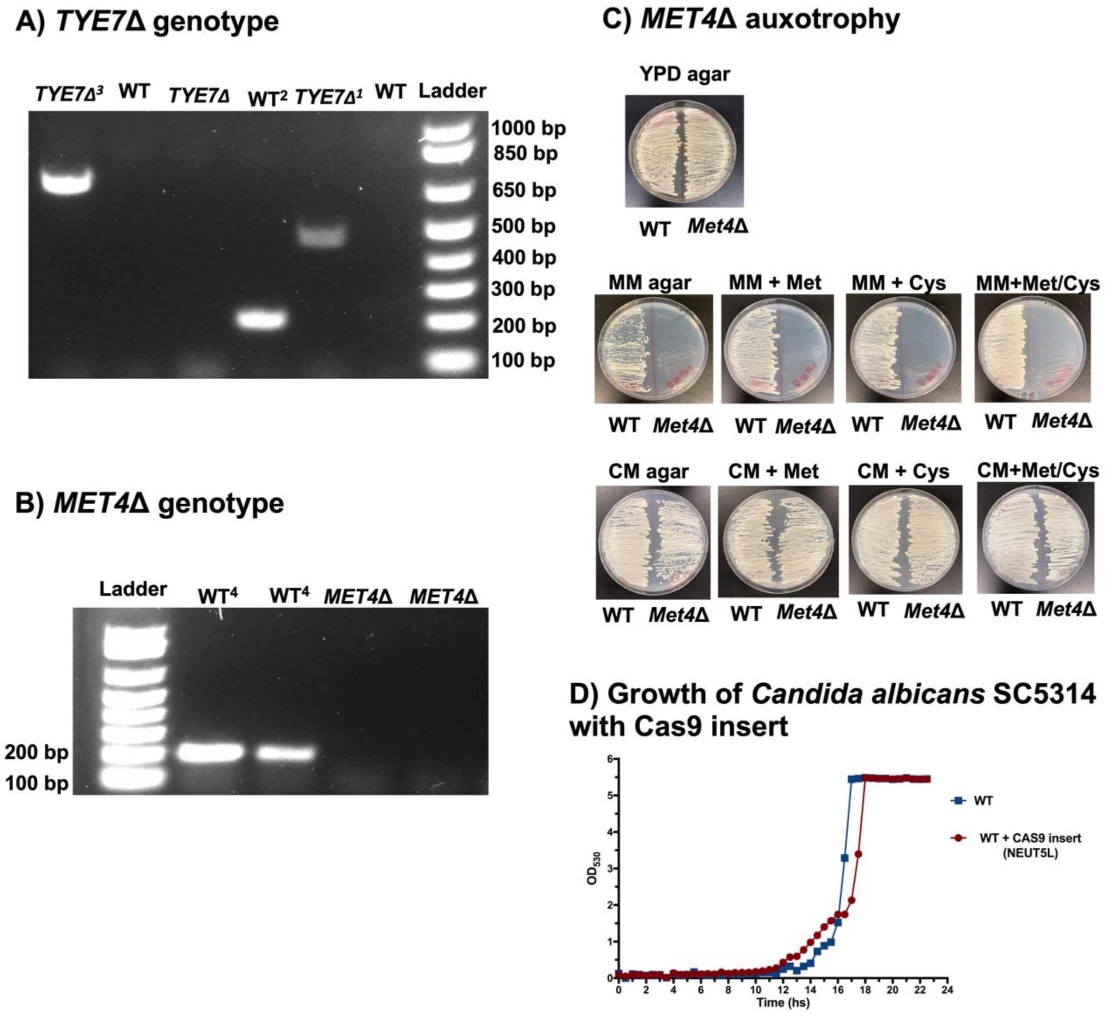

S3 Figure: Validation of the genotype of deletion mutants. **A)** Confirmation of generation of *TYE7*Δ mutant by PCR and gel electrophoresis where the *TYE7*Δ[1] band (~ 478 bp) confirmed the replacement of *TYE7* gene sequence with the gene drive cassette and the WT[2] band (~ 221 bp) confirmed the presence of *TYE7* gene in wild type. The deletion of the *TYE7* gene sequence in the *TYE7*Δ mutant (*TYE7*Δ[3] band; ~747 bp) confirmed the integration of Cas9 plasmid into *C. albicans* genome at the NEUT5L locus. **B)** Confirmation of *MET4*Δ genotype by PCR and gel electrophoresis where the WT[4] band (~ 224 bp) confirmed the presence of *MET4* gene in wild type and the deletion of *MET4* gene sequence in *MET4*Δ mutant. **C)** Growth of *C. albicans* SC5314 wild type and *MET4*Δ mutant in rich agar media (YPD), Minimal Media (MM), and Complete Minimal (CM) dropout media (synthetic mix minus methionine and cysteine) with or without addition of methionine and/or cysteine at 40 μg/mL final concentration. **D)** The integrated-plasmid NEUT5L did not show a defective effect on fungal fitness.
