# Supplementary material for: Combining Genome-Wide Gene Expression Analysis (RNA-seq) and a Gene Editing Platform (CRISPR-Cas9) to Uncover the Selectively Pro-oxidant Activity of Aurone Compounds Against Candida albicans
Source: Front Microbiol. 2021 Jul 15;12:708267. doi: 10.3389/fmicb.2021.708267 (PMC8319688; doi:10.3389/fmicb.2021.708267)
Supplement: Supplementary Figure 4 — Growth curves of SH1009 + FeCl3 treatments. [file Data_Sheet_4.pdf]

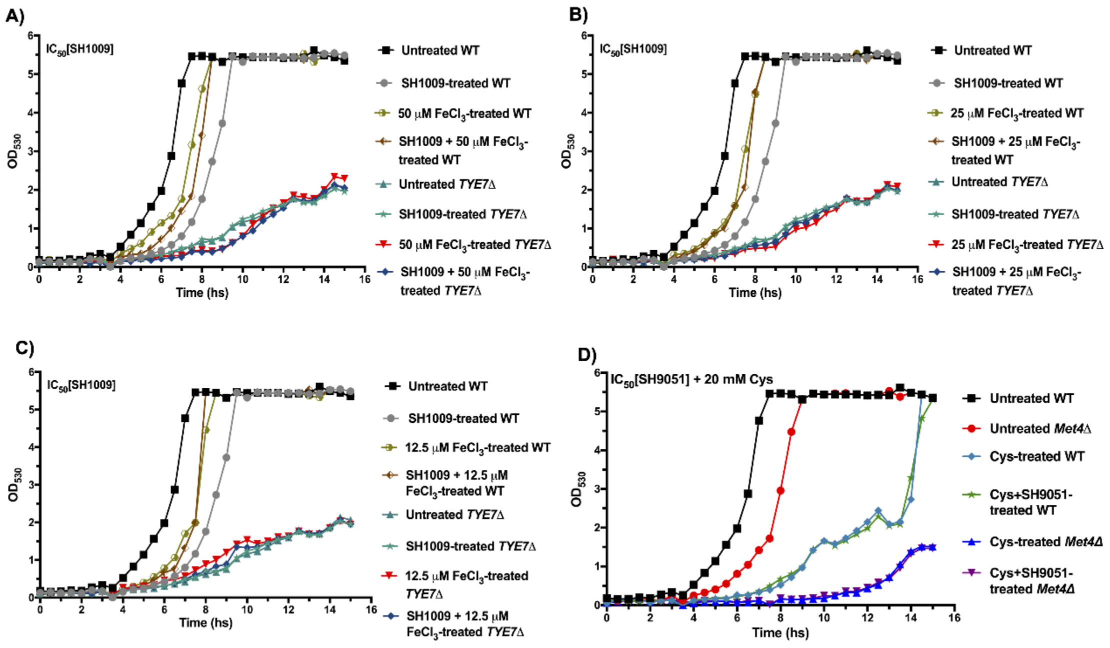

S4 Figure: (A, B, and **C)** The growth curves of *C. albicans* SC5314 wild-type strain and the *TYE7*Δ mutant under the IC_{50} concentration of aurone SH1009 (16 μM) supplemented with different concentrations of $FeCl_3$ (50, 25, and 12,5 μM). The growth conditions were carried out in YPD broth media using the Bioscreen C growth curve instrument and the OD_{530} reads were recorded at 30 min intervals for 24 h incubation at 30℃. **D)** The growth curves of *C. albicans* SC5314 wild-type strain and the *MET4*Δ mutant under the IC_{50} concentration of aurone SH9051 (90 μM) supplemented with 20 mM cysteine showed insignificant differences in growth for the wild type and mutant strains in contrast to treatment with 10 mM cysteine (in the main manuscript) that produced more susceptible growth for the *MET4*Δ mutant (**Figure 6F**).
