# Supplementary material for: Combining Genome-Wide Gene Expression Analysis (RNA-seq) and a Gene Editing Platform (CRISPR-Cas9) to Uncover the Selectively Pro-oxidant Activity of Aurone Compounds Against Candida albicans
Source: Front Microbiol. 2021 Jul 15;12:708267. doi: 10.3389/fmicb.2021.708267 (PMC8319688; doi:10.3389/fmicb.2021.708267)
Supplement: Supplementary Table 1 — Selectivity index of SH9051. [file Table_1.docx]

**Table 1:** The CC_50_ (cytotoxicity concentration of aurone SH9051 that results in 50% cell inhibition) and the selectivity index (SI) as a fraction between the CC_50_ for the human cell lines divided by the IC_50_ value (91.05 μM) against *C. albicans* SC5314.

| **Human cell line** | **CC_50_(µM)** | **SI** |
| --- | --- | --- |
| THP-1 (ATCC, TIB-202) | **111.2±3.4*** | **1.22** |
| HepG2 (ATCC, HB-8065) | **115.4±1.16** | **1.26** |
| A549 (ATCC, CCL-185) | **199.2±0.6** | **2.18** |

*Mean±SEM.
