# Supplementary material for: Combining Genome-Wide Gene Expression Analysis (RNA-seq) and a Gene Editing Platform (CRISPR-Cas9) to Uncover the Selectively Pro-oxidant Activity of Aurone Compounds Against Candida albicans
Source: Front Microbiol. 2021 Jul 15;12:708267. doi: 10.3389/fmicb.2021.708267 (PMC8319688; doi:10.3389/fmicb.2021.708267)
Supplement: Supplementary File 1 — RNA samples quantity and integrity. [file Presentation_1.pdf]

A)

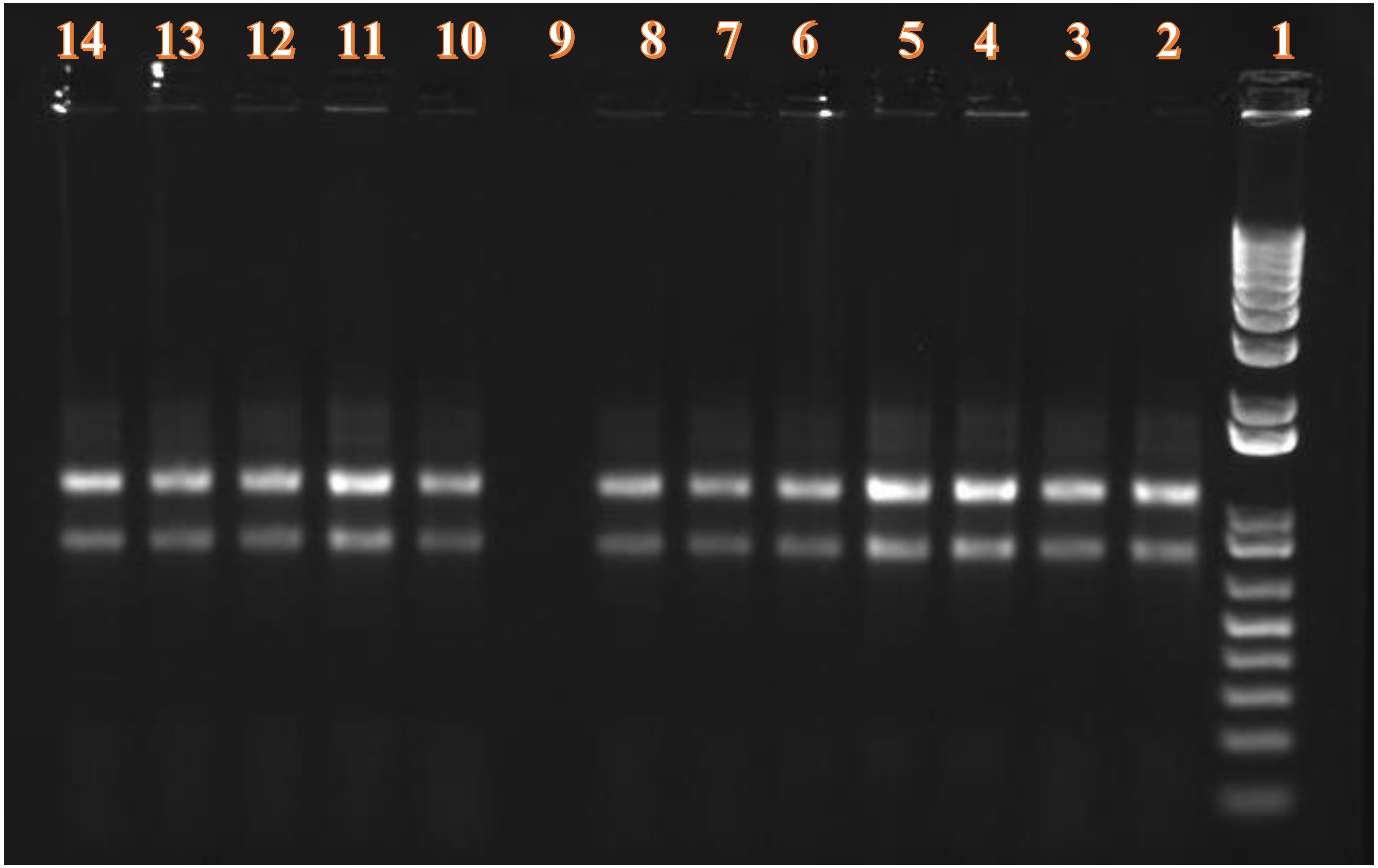

Lane 1: Ladder.

Lane 2: SH1009_1
Lane 3: SH1009_2
Lane 4: SH1009_3

Lane 7: SH9051_4
Lane 8: SH9051_5
Lane 10: SH9051_3

Lane 12: Untreated_1
Lane 13: Untreated_2
Lane 14: Untreated_3

B)

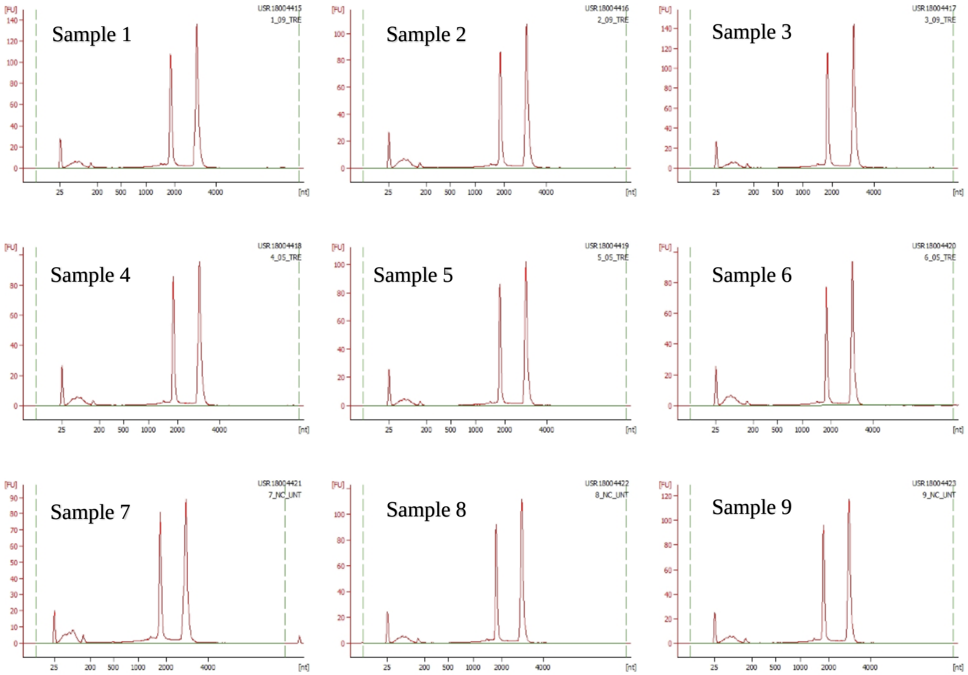

Sample Nr.	Sample Name	Amt. (µg)	$260/280_{ratio}$	RIN
1	SH1009_1	2.550	2.09	10
2	SH1009_2	1.890	2.06	10
3	SH1009_3	2.340	2.10	10
4	SH9051_1	1.750	2.08	9.9
5	SH9051_2	1.550	2.00	10
6	SH9051_3	1.630	2.03	10
7	Untreated_1	2.620	2.10	9.7
8	Untreated_2	1.770	2.00	10
9	Untreated_3	1.900	2.06	10

S1 File: Isolated RNA samples quantity, quality, and integrity: A) 1% agarose along with 1% v/v bleach TAE gel. Each well was loaded with 2 µL 6× DNA Loading dye + 10 µL of ≥ 1.5 µg of total RNA isolated from SH1009- and SH9051-treated, and untreated *Candida albicans* SC5314 cells. All samples were run for ~30 min at a constant 120V in 1% TAE buffer. The presence of clean 2:1 ratios of 28S and 18S ribosomal RNA (rRNA) bands suggested intact RNA samples without DNA contamination (1). **B)** Agilent Bioanalyzer analysis of RNA integrity. The fluorescence signal generated by the Agilent Bioanalyzer instrument indicated intact RNA samples with RIN (RNA Integrity Number) values range from 9.7 to 10 with two discrete peaks corresponding to 18S and 28S rRNA bands on the gel.

Reference:

1. Aranda PS, LaJoie DM, Jorcyk CL. Bleach gel: a simple agarose gel for analyzing RNA quality. Electrophoresis. 2012;33(2):366-9.
